# Mutant analysis in the nonlegume *Parasponia andersonii* identifies NIN and NF‐YA1 transcription factors as a core genetic network in nitrogen‐fixing nodule symbioses

**DOI:** 10.1111/nph.16386

**Published:** 2020-01-30

**Authors:** Fengjiao Bu, Luuk Rutten, Yuda Purwana Roswanjaya, Olga Kulikova, Marta Rodriguez‐Franco, Thomas Ott, Ton Bisseling, Arjan van Zeijl, Rene Geurts

**Affiliations:** ^1^ Laboratory of Molecular Biology Department of Plant Science Wageningen University Droevendaalsesteeg 1 6708PB Wageningen the Netherlands; ^2^ Center of Technology for Agricultural Production Agency for the Assessment and Application of Technology (BPPT) 10340 Jakarta Indonesia; ^3^ Cell Biology Faculty of Biology University of Freiburg 79104 Freiburg Germany

**Keywords:** evolution, intracellular infection, NF‐YA1, NODULE INCEPTION (NIN), nodulation, *Parasponia*, rhizobium

## Abstract

● Nitrogen‐fixing nodulation occurs in 10 taxonomic lineages, with either rhizobia or *Frankia* bacteria. To establish such an endosymbiosis, two processes are essential: nodule organogenesis and intracellular bacterial infection. In the legume–rhizobium endosymbiosis, both processes are guarded by the transcription factor NODULE INCEPTION (NIN) and its downstream target genes of the NUCLEAR FACTOR Y (NF‐Y) complex.
● It is hypothesized that nodulation has a single evolutionary origin *c*. 110 Ma, followed by many independent losses. Despite a significant body of knowledge of the legume–rhizobium symbiosis, it remains elusive which signalling modules are shared between nodulating species in different taxonomic clades. We used *Parasponia andersonii* to investigate the role of *NIN* and *NF‐YA* genes in rhizobium nodulation in a nonlegume system.
● Consistent with legumes, *P. andersonii PanNIN* and *PanNF‐YA1* are coexpressed in nodules. By analyzing single, double and higher‐order CRISPR‐Cas9 knockout mutants, we show that nodule organogenesis and early symbiotic expression of *PanNF‐YA1* are PanNIN‐dependent and that PanNF‐YA1 is specifically required for intracellular rhizobium infection.
● This demonstrates that *NIN* and *NF‐YA1* have conserved symbiotic functions. As *Parasponia* and legumes diverged soon after the birth of the nodulation trait, we argue that NIN and NF‐YA1 represent core transcriptional regulators in this symbiosis.

## Introduction

Nitrogen (N) is an essential element for plant growth. To cope with N limitation, some plant species engage with N_2_‐fixing rhizobium or *Frankia* bacteria. These bacteria colonize cells of specialized root organs, called nodules. Inside nodule cells, the bacteria convert atmospheric N into ammonium which can be exploited by the plant. Plant species capable of forming N_2_‐fixing nodules all belong to one of the four orders, Fabales, Fagales, Cucurbitales and Rosales, that together form the so‐called N‐fixing clade (Soltis *et al*., 1995; Doyle, 2011). Within this clade, nodulation is limited to 10 lineages, of which eight nodulate with *Frankia* and two with rhizobia (Geurts *et al*., 2012). The nodulating lineages within the N‐fixing clade are interspersed among tens of nonnodulating lineages. The current hypothesis is that this scattered distribution originates from a single evolutionary gain of nodulation in the ancestor to the N_2_‐fixing clade, and subsequent loss of this trait in many descending species (Griesmann *et al*., 2018; van Velzen *et al*., 2018, 2019). Such a scenario implies that the nodulation trait in all 10 lineages is based on conserved genetic networks.

Rhizobium‐induced nodulation occurs in two lineages; *Parasponia* (Cannabaceae, Rosales) and legumes (Fabaceae, Fabales). These lineages diverged > 100 Ma and even though the capacity to live in endosymbiosis with diazotrophic bacteria may have been the result of a shared evolutionary event, *Parasponia* and legumes probably acquired rhizobium as a microsymbiont in parallel (van Velzen *et al*., 2018, 2019). The molecular and genetic aspects of rhizobium‐induced nodulation have been extensively studied in a number of legume species, for example pea (*Pisum sativum*), *Medicago truncatula* and *Lotus japonicus*, whereas some data are also available for *Parasponia*. To initiate symbiosis, most rhizobium bacteria excrete lipo‐chitooligosaccharide (LCO) signals that are perceived by plant LysM‐type receptor kinases (Lerouge *et al*., 1990; Dénarié *et al*., 1996; Limpens *et al*., 2003; Madsen *et al*., 2003; Radutoiu *et al*., 2003; Op den Camp *et al*., 2011). LCO perception activates the so‐called ‘common symbiosis signalling pathway’, which is coopted from arbuscular mycorrhizal symbiosis (Oldroyd, 2013). Downstream of the common symbiosis signalling pathway, it culminates in the activation of a suite of transcriptional regulators (Soyano & Hayashi, 2014). Among these are NODULE INCEPTION (NIN) and its downstream targets of the NUCLEAR FACTOR Y (NF‐Y) complex that are essential for nodule organogenesis and rhizobium infection and among the first genes transcriptionally induced (Schauser *et al*., 1999; Combier *et al*., 2006; Marsh *et al*., 2007; Soyano *et al*., 2013; Rípodas *et al*., 2014; Vernié *et al*., 2015).

NUCLEAR FACTOR Y complexes are heterotrimeric transcription factors composed of the NF‐YC, NF‐YB and NF‐YA subunits, of which the latter determines the DNA‐binding specificity (Baudin *et al*., 2015; Myers & Holt, 2018). In plants, each of these subunits is encoded by a small family and in legumes several NF‐Y‐encoding genes display a nodule‐enhanced expression profile (Laloum *et al*., 2013; Baudin *et al*., 2015). Mutant analysis in *L. japonicus* and *M. truncatula* revealed that *NF‐YA1* is required for nodule development (Combier *et al*., 2006; Soyano *et al*., 2013; Laloum *et al*., 2014; Laporte *et al*., 2014; Hossain *et al*., 2016). In *L. japonicus nf‐ya1* mutants, most nodules do not progress beyond the primordial stage, whereas *M. truncatula nf‐ya1* mutants develop nodules of variable size, but all remain substantially smaller than wild‐type nodules (Combier *et al*., 2006; Hossain *et al*., 2016). The latter is most probably a result of disturbed formation of the nodule apical meristem (Combier *et al*., 2006; Laloum *et al*., 2014; Laporte *et al*., 2014; Xiao *et al*., 2014). Besides problems in nodule organogenesis, *M. truncatula nf‐ya1* mutants are also affected in the formation of intracellular infection threads (Laporte *et al*., 2014). These infection threads initiate at the tip of a root hair and function to guide rhizobium bacteria to the underlying nodule primordium, which is formed in the root cortex. In *M. truncatula nf‐ya1* mutants, infection thread progression is hampered and infection thread growth is frequently arrested in the epidermal layer (Laporte *et al*., 2014). In *L. japonicus*, *Ljnf‐ya1* knockdown lines display only a very weak infection phenotype (Soyano *et al*., 2013; Hossain *et al*., 2016). Taken together, this shows that in legumes *NF‐YA* genes function during rhizobia infection and nodule organogenesis.

In legumes, *NIN* is among the first genes transcriptionally activated upon rhizobium LCO signalling, which is acting downstream of the common symbiosis signalling pathway, and is essential as well as sufficient to initiate nodule organogenesis (Schauser *et al*., 1999; Borisov *et al*., 2003; Marsh *et al*., 2007; Soyano *et al*., 2013). NIN belongs to a small family of NIN‐Like proteins (NLPs), of which, in *Arabidopsis thaliana*, several members are involved in nitrate signalling (Schauser *et al*., 2005; Castaings *et al*., 2009; Konishi & Yanagisawa, 2013). Orthologues of *NIN* are found across eudicots, but within the N‐fixation clade functional copies of this gene have been repeatedly lost from the genomes of nonnodulating species (Griesmann *et al*., 2018; van Velzen *et al*., 2018). This suggests that within the N_2_‐fixation clade, NIN predominantly performs a nodulation‐specific function. The first indication that this is indeed the case is obtained from *Agrobacterium tumefaciens*‐mediated stable transformation knockdown studies in *Casuarina glauca*, which resulted in a reduced nodulation efficiency when inoculated with *Frankia* (Clavijo *et al*., 2015). However, such functional studies to prove that NIN – and its subsequent *NF‐YA* target genes – has key symbiotic roles in nodulating lineages other than legumes remain scarce.

We aimed to use *Parasponia* to investigate the extent to which NIN and NF‐YA transcriptional regulators have conserved functions in root nodule formation. Previous studies have shown that *NIN* and *NF‐YA1* are transcriptionally induced in *Parasponia andersonii* nodules (van Velzen *et al*., 2018). By creating a series of CRISPR‐Cas9 knockout mutants, we provide evidence that *PanNIN* is essential for nodule initiation in the nonlegume *P. andersonii*. Furthermore, we show that *PanNF‐YA1* is specifically required for intracellular rhizobium infection, whereas nodule organogenesis is controlled by a genetically redundant network of *NF‐YA* genes. Taken together, this suggests that *NIN* and *NF‐YA1* are part of a core genetic network essential for rhizobium symbiosis in legumes and nonlegume species.

## Materials and Methods

### Plant materials and growth conditions

All experiments were done using *P. andersonii* WU1.14 (van Velzen *et al*., 2018; Wardhani *et al*., 2019). Plants were maintained as described previously (van Zeijl *et al*., 2018; Wardhani *et al*., 2019). Young plantlets for nodulation assays were vegetatively propagated *in vitro*, rooted, and inoculated with *Mesorhizobium plurifarium* BOR2 at an OD_600_ = 0.03 (van Velzen *et al*., 2018; van Zeijl *et al*., 2018; Wardhani *et al*., 2019). For early induction of symbiotic genes, we made use of *Rhizobium tropici* CIAT899 transformed with pMP604 (OD_600_ = 0.03–0.05) (Martínez *et al*., 1985; Spaink *et al*., 1989). Nodulation efficiencies were calculated by determining the average nodule number per plant. Nodule size estimates were determined by measuring the two‐dimensional nodule surface area using imagej (Abràmoff *et al*., 2004). Comparisons were made based on the average nodule size per plant using at least four replicate plants. Acetylene reductase assays (ARAs) were conducted as described previously (van Velzen *et al*., 2018). Mycorrhization experiments were conducted using 250 spores of *Rhizophagus irregularis* strain DOAM197198, as described previously (van Velzen *et al*., 2018; Wardhani *et al*., 2019).

### Lateral root growth assay

Similar‐sized rooted plantlets were grown on EKM‐plates (1% Daishin agar) (Duchefa, Haarlem, the Netherlands) in between two cellophane layers cut to 12 × 8 cm (gel drying frames; Sigma Aldrich) (van Velzen *et al*., 2018; Wardhani *et al*., 2019). Plants were grown vertically at a 60º angle for 20 d at 28°C, in a 16 h : 8 h, light : dark regime. The main roots were determined as all roots directly attached to the shoot that were present at the start of the experiment. Per plantlet, root length and lateral root number per root were determined. Total ‘main’ root length per shoot and lateral root density in lateral roots mm^–1^ root were plotted per plant. Statistical testing was based on a Mann–Whitney *U*‐test with a significance level of *P* < 0.05.

### Vectors and constructs

Single‐guide RNAs (sgRNAs) were designed using the ‘Find CRISPR Targets’ function implemented in geneious 9.1.5 (Biomatters, Auckland, New Zealand) and subsequently checked against the *P. andersonii* genome for high‐identity off‐targets. To mutate genes, up to three sgRNAs were used to target either the first or the second coding exon (Supporting Information Table S1). Selected sgRNAs were amplified using sequence‐specific forward primers and a universal reverse primer (Table S2), using Addgene plasmid no. 46966 as template (Nekrasov *et al*., 2013). Constructs for CRISPR/Cas9‐mediated mutagenesis were assembled as described previously (van Zeijl *et al*., 2018; Wardhani *et al*., 2019). To allow golden gate cloning of β‐glucuronidase (GUS) reporter constructs, the *Bpi*I and *Bsa*I restriction sites in putative promoter sequences of *PanNF‐YA1, PanNF‐YA3* and *PanNF‐YA6* were mutated by introducing single nucleotide substitutions (Engler *et al*., 2014). The putative promoter sequences are provided in Table S3.

The Gene Identifiers and GenBank accession nos. of the used *P. andersonii* genes are: *PanNIN*: PanWU01x14_111140, PON66248.1; *PanNF‐YA1:* PanWU01x14_284830, PON42093.1; *PanNF‐YA3*: PanWU01x14_246880, PON47071.1; and *PanNF‐YA6*: PanWU01x14_192330, PanWU01x14_192330.

### Plant transformation

*Agrobacterium tumefaciens*‐mediated transformation and genotyping were done as previously described (van Zeijl *et al*., 2018; Wardhani *et al*., 2019). Primers used for genotyping are listed in Table S2. For promoter‐GUS reporter studies, we investigated five independent lines for each construct.

### Histochemical analysis, microtome sectioning and microscopy

Root and nodule samples of the *PanNF‐YA_pro_:GUS* lines were incubated in GUS buffer (3% (w/v) sucrose, 10 mM EDTA, 2 mM k‐ferrocyanide, 2 mM k‐ferricyanide, and 0.5 mg ml^−1^ 5‐bromo‐4‐chloro‐3‐indolyl‐β‐d‐glucuronic acid, cyclohexylammonium salt (X‐Gluc) in 0.1 M phosphate buffer (pH 7.2)) at 37°C for 2 and 5 h, respectively. For whole mount sections, GUS‐stained samples were embedded in 6% low‐melting‐point agarose (in PBS). Sections (70 µm thick) were made using a vibratome, and were imaged using Nomarski microscopy. For plastic sections, root segments and nodules were fixed in 4% paraformaldehyde (w/v), 5% glutaraldehyde (v/v) in 50 mM phosphate buffer (pH 7.2) at 4°C for 24 h. Subsequently, the samples were dehydrated using an ethanol series and embedded in Technovit 7100 (Heraeus Kulzer, Hanau, Germany) according to the manufacturer's instructions. Semithin sections were cut using a Leica Ultracut microtome (Leica Microsystems, Wetzlar, Germany) to 4 µm thickness for nodules formed on CRISPR mutant lines and 7 µm thickness for GUS‐stained samples. Sections were stained with 0.05% Toluidine Blue or 0.1% Rethudium Red. Images were photographed using a Leica DM5500B microscope equipped with a DFC425C camera (Leica Microsystems). Samples for electron microscopy were fixed in MTSB buffer (Pasternak *et al*., 2015) containing 2.5% glutaraldehyde, postfixed in aqueous 1% OsO_4_ solution, and stained *in bloc* with 1% uranyl acetate. After dehydration in increasing EtOH concentrations, samples were embedded in epoxy resin. Ultrathin (70 nm) sections were poststained with 2% uranyl acetate and observed in a Philips CM‐10 TEM (Thermo Fisher Scientific, Hillsboro, OR, USA). Images were taken using a Gatan BioScan 792 camera (Gatan, Pleasanton, CA, USA).

### *In situ* hybridization

*Parasponia andersonii* nodules were fixed with 4% paraformaldehyde, 3% glutaraldehyde in 50 mM phosphate buffer (pH 7.4) and embedded in paraffin (Paraplast X‐tra; Leica Biosystems, Wetzlar, Germany). Root sections of 7 μm were prepared using an RJ2035 microtome (Leica Microsystems). RNA *in situ* hybridization (ISH) was conducted using Invitrogen ViewRNA™ ISH Tissue 1‐ Plex Assay kits (Thermo Fisher Scientific, Waltham, MA USA) according to a protocol previously developed for *M. truncatula* (Kulikova *et al*., 2018). In short, mRNA detection is based on branched (b)DNA signal amplification technology. A mRNA probe set contains *c*. 20 synthetic adjacent oligonucleotide pairs. Each oligonucleotide is composed of a 20 bp primary sequence to target the sequence of interest and a secondary extended sequence serving as a template for hybridization of a preamplifier oligonucleotide. The preamplifier can hybridize to two adjacent probes. An additional sequence of the preamplifier is designed to hybridize to multiple bDNA amplifier molecules that create a branched structure. Finally, alkaline phosphatase (AP)‐labelled oligonucleotides, which are complementary to bDNA amplifier sequences, bind to the bDNA molecule by hybridization. By adding Fast Red substrate (ThermoFisher Scientific), red punctuated precipitates are formed that can be detected by light microscopy. RNA ISH probe sets were designed and synthesized on request by ThermoFisher Scientific. Catalogue numbers of probes are VF1‐6000380 for *PanNIN*, VF1‐6000400 for *PanNF‐YA1*, VF1‐6000767 for *PanNF‐YA3*, and VF‐6000766 for *PanNF‐YA6*. Images were taken with an DM5500B microscope equipped with a DFC425C camera (Leica Microsystems).

### Phylogenetic reconstruction

Protein sequences of *L. japonicus* (Lj3.0, *Lotus* Base (REF; Mun *et al*., 2016); Sato *et al*., 2008), *Glycine max* (Wm82.a2.v1; Sato *et al*., 2008; Schmutz *et al*., 2010), *Phaseolus vulgaris* (Pvulgaris v.2.1; Schmutz *et al*., 2014), *Morus notabilis* (Genbank ATGF00000000.1; He *et al*., 2013), *Prunus persica* (Ppersica v.2.1; International Peach Genome Initiative *et al*., 2013) *Fragaria vesca* (Fvesca v.1.1; Shulaev *et al*., 2011) were retrieved from Phytozome 12 (http://phytozome.jgi.doe.gov/), unless stated otherwise. *Casuarina glauca* and *Datisca glomerata* assemblies were downloaded and set up as custom Blast database in geneious 8.1.9 (Griesmann *et al*., 2018; van Velzen *et al*., 2018). Sequences from diploid Peanut *Arachis duranensis* were retrieved from NCBI (Bertioli *et al*., 2016). Protein sequences of *P. andersonii* (PanWU01x14) and *Trema orientalis* (TorRG33x02) were obtained from http://www.parasponia.org (van Velzen *et al*., 2018; Holmer *et al*., 2019). These sequences were mined using sequences from *A. thaliana* (Tair10; Lamesch *et al*., 2012) and *M. truncatula* (Mt4.0v1; Young *et al*., 2011; Tang *et al*., 2014). Protein sequences were aligned using mafft v.7.017 (parameter settings: algorithm, auto; scoring matrix, Blosum62; gap open penalty, 1.53; offset value, 0.123; Katoh *et al*., 2002; Katoh & Standley, 2013; Table S4) implemented in geneious 8.1.9. Bayesian phylogeny was reconstructed using mrbayes 3.2.6. (Ronquist & Huelsenbeck, 2003) implemented in geneious 8.1.9. (parameter settings: rate matrix, poisson; rate variation, gamma; gamma categories, 4; chain length, 5100 000; heated chains, 4; heated chain temp, 0.2; subsampling freq, 1000; burn‐in length, 100 000; random seed, 8681). Midpoint rooting was applied for better tree visualization using figtree v.1.4.2. (http://tree.bio.ed.ac.uk/software/figtree).

### RNA isolation and qRT‐PCR analysis

RNA was isolated from snap‐frozen root segments of *c*. 0.5 cm, which includes the elongation zone and the newly formed differentiation zone. cDNA was prepared from 1 μg of total RNA using the i‐script cDNA synthesis kit (Bio‐Rad), following the manufacturer's instructions. Ten microlitre quantitative reverse transcription polymerase chain reaction (qRT‐PCR) reactions were set up using 2× iQ SYBR Green Supermix (Bio‐Rad) and 5 ng template DNA. Quantification was performed using a CFX Connect optical cycler, according to the manufacturer's protocol (Bio‐Rad). Normalization was performed based on the stably expressed reference gene *ELONGATION FACTOR 1α* (*PanEF1α*; van Zeijl *et al*., 2018). Primers used for qPR‐PCR analysis are listed in Table S2.

## Results

### *P. andersonii* NIN and NF‐YA1 are coexpressed during nodule formation

Previously conducted transcriptome studies revealed that *PanNIN* and *PanNF‐YA1* have a nodule‐enhanced expression profile in *P. andersonii* (van Velzen *et al*., 2018). To obtain a first insight into the spatiotemporal expression pattern of both genes, we conducted promoter reporter and/or ISH experiments. To this end, a 3.8 kb sequence upstream of the translational start site of *PanNF‐YA1*, containing the putative promoter sequence and the 5'‐UTR that includes the first intron, was fused to a GUS‐encoding sequence. The resulting construct was introduced into the *P. andersonii* genome using *A. tumefaciens*‐mediated stable transformation (van Zeijl *et al*., 2018). Five lines were selected, for which we compared the GUS reporter activity under symbiotic and nonsymbiotic conditions. Four of these lines yielded comparable results, and therefore one of these lines (line 1E5) was selected for detailed characterization.

**Figure 1:**
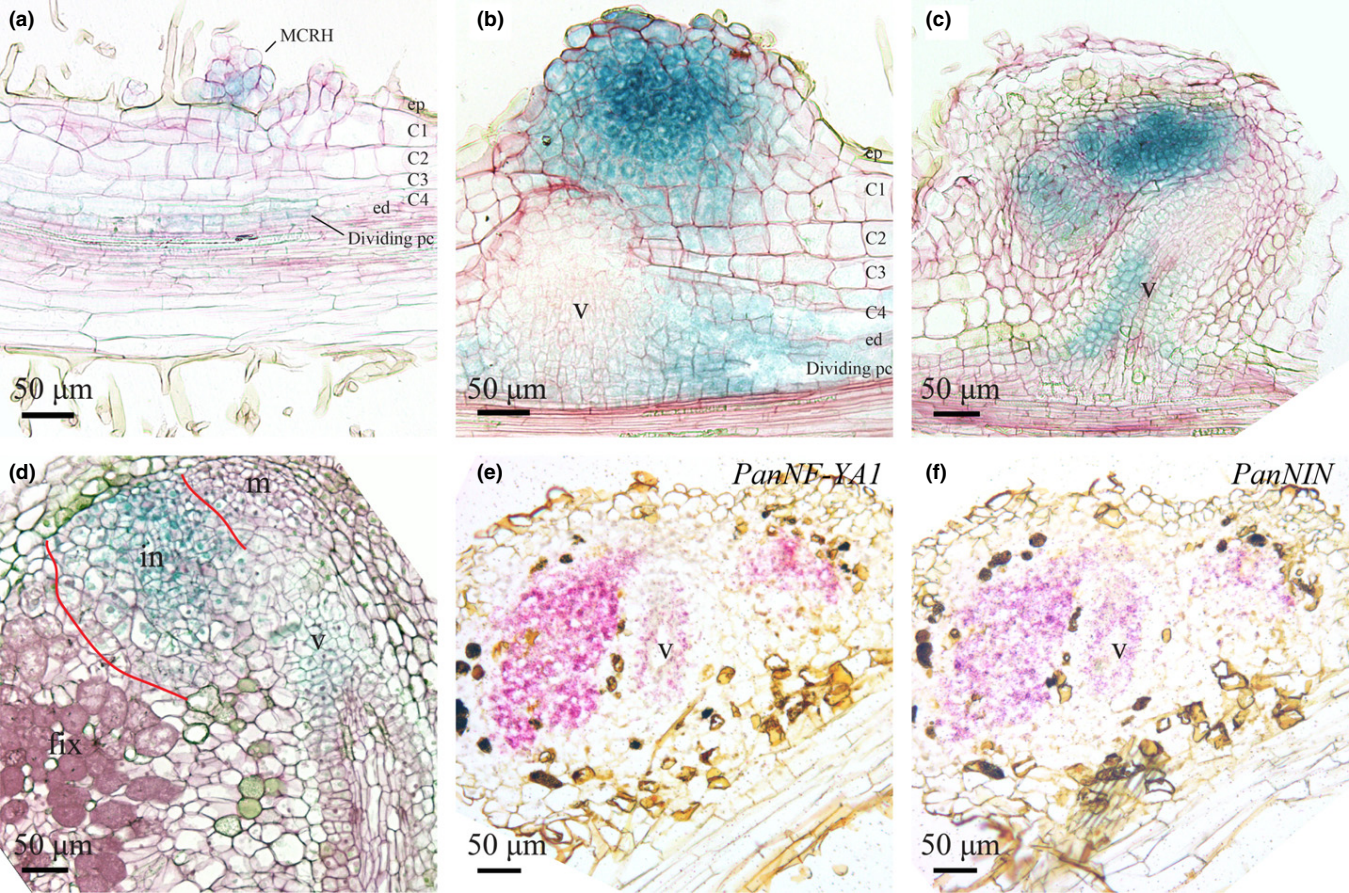
Spatiotemporal expression pattern of *PanNF‐YA1* and *PanNIN* in developing *Parasponia andersonii* root nodules. (a–d) Spatiotemporal expression pattern of *PanNF‐YA1*pro:GUS in nodules of different developmental stages. (e, f) Spatiotemporal expression pattern of *PanNF‐YA1* and *PanNIN* visualized by *in situ* hybridization on consecutive sections of a young *P. andersonii* nodule primordium. (a) *PanNF‐YA1*pro:GUS activity in clustered root hairs that are associated with dividing epidermal, outer cortical and pericycle cells. (b) P*anNFYA1*pro:GUS activity in a young but not yet intracellularly infected nodule and in the pericycle‐derived cells flanking the developing nodule vasculature. (c) *PanNF‐YA1*pro:GUS activity in the infection zone of young nodules, and in the basal part of the nodule vasculature. (d) *PanNF‐YA1*pro:GUS activity in a mature nodule is restricted to the infection zone (marked with red lines) and nodule vasculature. (e, f) *PanNF‐YA1* (e) and *PanNIN* (f) transcripts are detected in the infection zone and nodule vasculature by *in situ* hybridization on consecutive sections. MCRH, multicellular root hairs; ep, epidermis; C1‐C4, first to fourth cortical cell layer; ed, endodermis; pc, pericycle; m, nodule meristem; in, infection zone; fix, fixation zone; v, nodule vasculature. In (a)–(d), sections (7 µm) were counterstained with Ruthenium Red. Nodules were isolated 4 wk post‐inoculation with *Mesorhizobium plurifarium* BOR2.

Under sterile conditions, activity of the *PanNF‐YA1_pro_*:*GUS* was observed around the vasculature of differentiated root tissue (Fig. S1a,b). Root sections revealed that GUS staining is restricted to the pericycle cells opposite to the protoxylem, but absent from lateral root primordia (Fig. S1b–d). In *M. truncatula*, similar promoter‐GUS studies using a 2.2 kb upstream region revealed that *MtNF‐YA1* is induced in root hairs of the preinfection zone and in the root pericycle upon rhizobium inoculation (Laporte *et al*., 2014; Liu *et al*., 2019). We questioned whether this is also the case for *P. andersonii*. To determine this, transgenic plantlets expressing the *PanNF‐YA1_pro_*:*GUS* reporter were grown *in vitro* on N‐poor medium (0.375 mM NH_4_NO_3_) and inoculated with *Mesorhizobium plurifarium* BOR2. In contrast to legumes like *M. truncatula* and *L. japonicus*, *Parasponia* species are not infected via curled root hairs. Instead, rhizobia enter apoplastically via cracks that are formed upon cell divisions in the epidermis and outer cortex and only infect intracellularly when a nodule primordium is formed (Lancelle & Torrey, 1984, 1985). At 2 d post‐inoculation (dpi), *PanNF‐YA1_pro_*:*GUS* activity was observed in epidermal and cortical cells located just above the root elongation zone (Fig. S1e). *PanNF‐YA1_pro_:GUS* is active in clumps of multicellular root hairs and adjacent cortical cells as well as dividing pericycle‐derived cells (Figs 1a, S1f,g). The formation of multicellular root hairs is one of the earliest responses associated with nodule initiation in *Parasponia* species and is not observed in noninoculated roots (Lancelle & Torrey, 1984, 1985). In young nodule primordia that are visible as small bumps on the root (5 dpi), the *PanNF‐YA1_pro_*:*GUS* reporter was highly active in clusters of dividing cells (Fig. 1b). Additionally, activity was observed in dividing pericycle cells that flank the developing nodule vascular bundle (Fig. 1b). In young nodules, *PanNF‐YA1_pro_*:*GUS* activity is observed in the central region of the nodule lobes, where intracellular infection by rhizobium will occur (Fig. 1c). In mature nodules, *PanNF‐YA1_pro_*:*GUS* activity was mostly confined to the infection zone (Fig. 1d). Additionally, weaker activity is observed in the cell layers surrounding the nodule vascular bundle (Fig. 1c,d). Taken together, the expression pattern of the *PanNF‐YA1_pro_*:*GUS* reporter suggests a symbiotic role of *PanNF‐YA1*.

Next, we determined whether *PanNF‐YA1* is coexpressed with *PanNIN* in *P. andersonii* nodules. As regulation of *NIN* in legumes has been shown to be highly complex and determined by distant *cis*‐regulatory elements (Heckmann *et al*., 2011; Kosuta *et al*., 2011; Popp & Ott, 2011; Soyano *et al*., 2014; Yoro *et al*., 2014; Liu *et al*., 2019), we decided to use RNA ISH. This method showed the accumulation of the *PanNIN* transcripts in the central region of the lobes where rhizobium infection will take place and in the pericycle/endodermis of the vasculature of young nodules (Fig. 1f). ISH on a consecutive section of the same nodule showed that the *PanNF‐YA1* transcripts are present in the same cells as *PanNIN* (Fig. 1e,f), and that transcript accumulation is consistent with the activity of the *PanNF‐YA1_pro_*:*GUS* reporter in a nodule of a similar developmental stage (Fig. 1c). Therefore, we conclude that *PanNIN* and *PanNF‐YA1* are coexpressed in young nodules.

### PanNIN is essential for nodule formation and symbiotic expression of PanNF‐YA1

**Figure 2:**
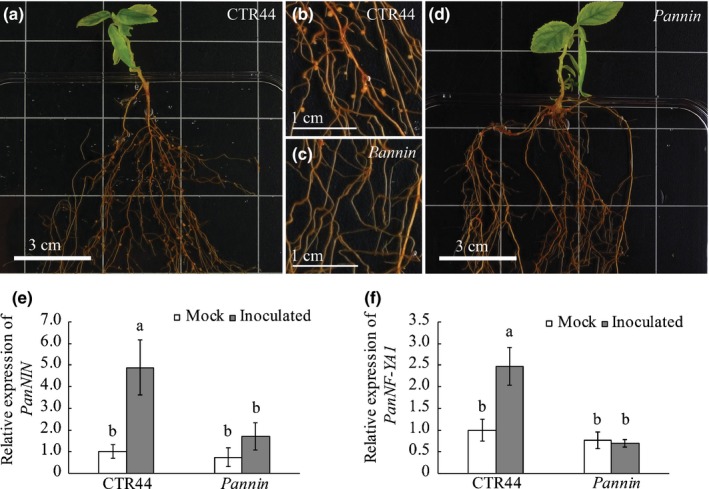
Symbiotic phenotype of the *Parasponia andersonii nin* mutant. Shown are (a, b) a transgenic control (CTR44) and (c, d) a *Pannin* knockout mutant (line B3) at 4 wk post‐inoculation with *Mesorhizobium plurifarium* BOR2. Note that nodules are present on roots of the control (a, b), but not on *Pannin* mutant roots (*n* = 50) (c, d). These images are representative results obtained from three independent experiments, with > 20 plants combined for each line. (e) Relative expression of *PanNIN* in noninoculated and inoculated transgenic control (CTR44) and *Pannin* mutant (line B3) roots. (f) Relative expression of *PanNF‐YA1* in noninoculated and inoculated transgenic control (CTR44) and *Pannin* mutant (line B3) roots. RNA was isolated from root segments encompassing the elongation and part of the differentiation zone at 1 d post‐inoculation (dpi) with *Rhizobium tropici* CIAT899 pMP604. Data represent means of two independent experiments with a total of five biological replicates each ± SE. Data were normalized against the mock‐treated CTR44 sample. Different letters indicate statistical significance (Student's *t*‐test, *P* < 0.05).

To determine whether *PanNIN* is essential for nodule formation in *P. andersonii*, we created *Pannin* knockout mutants using CRISPR/Cas9‐mediated mutagenesis. The *NIN* gene in *Parasponia* species produces two alternative transcript variants: using a transcriptional initiation site at the 5'‐end of the gene (*PanNIN.1*); and an alternative transcriptional initiation site for *PanNIN.2* located in the second intron of the gene (Fig. S2a; van Velzen *et al*., 2018). Quantification of RNAseq reads revealed that both transcripts are expressed in roots, whereas only expression of the long transcript (*PanNIN.1*) encoding a canonical NIN protein is enhanced in nodules (Fig. S2b). Therefore, we decided to create CRISPR‐Cas9 mutants exclusively mutated in the long *NIN* transcript (*PanNIN.1*). Two knockout mutant lines (named B1 and B3) were obtained by targeting the first coding‐exon using three sgRNAs (Fig. S2c). These mutants contain premature stop codons at amino acid positions 90 (line B1) and 70 (line B3), respectively (Fig. S2d). Inoculation with *M. plurifarium* BOR2 showed that both lines are unable to form root nodules or even nodule primordia (Fig. 2c,d), whereas a transgenic control line (CTR44) was well nodulated (Fig. 2a,b). This demonstrates that the *PanNIN.1* transcript is essential for nodule organogenesis in *P. andersonii*.

To determine whether rhizobium‐induced *PanNF‐YA1* expression is dependent on a functional PanNIN.1 protein, we conducted qRT‐PCR experiments. Root RNA was isolated from a *c*. 0.5 cm region encompassing part of the root elongation and differentiation zone at 1 dpi with a compatible rhizobium strain that harbours a dominant active NodD protein that transcriptionally activates LCO biosynthesis genes (*Rhizobium tropici* CIAT899 pMP604; Spaink *et al*., 1989; Op den Camp *et al*., 2012; Fig. S3). In roots of transgenic control line CTR44, expression of *PanNIN.1* and *PanNF‐YA1* was induced five‐ and 2.5‐fold following inoculation, respectively (Fig. 2e,f). By contrast, such induction of *PanNF‐YA1* is not observed in *Pannin* mutant roots (Fig. 2e,f). This indicates that the early symbiotic induction of *PanNF‐YA1* is downstream of PanNIN.1.

### PanNF‐YA1 is essential for rhizobium intracellular infection

To determine the symbiotic role of *PanNF‐YA1*, we mutated this gene using CRISPR/Cas9. To this end, sgRNAs were designed that target the first coding‐exon of *PanNF‐YA1* (Table S1; Fig. S4a). This allowed the isolation of *Pannf‐ya1* knockout mutant line (Fig. S4b).

We noted that *Pannf‐ya1* mutant shoots were somewhat more difficult to root (Fig. S5a,b), a phenotype we did not observe with transgenic control or *Pannin* mutant shoots. As it was reported previously that *NF‐YA1* orthologous genes may function in root growth and lateral root formation (Soyano *et al*., 2013; Sorin *et al*., 2014), we quantified root development in the *Pannf‐ya1‐1* mutant line and transgenic control. This revealed that the *Pannf‐ya1‐1* mutant formed less lateral roots when compared with transgenic controls (Fig. S5c–f).

To determine the nodulation phenotype, the *Pannf‐ya1‐1* mutant line plants were grown in Perlite and inoculated with *M. plurifarium* BOR2. This showed that *Pannf‐ya1‐1* can be nodulated at least as efficiently as control plants (Fig. S6a). However, quantification of Nitrogenase activity using the ARA indicated that *Pannf‐ya1‐1* nodules are unable to fix N_2_ (Fig. S6b).

**Figure 3:**
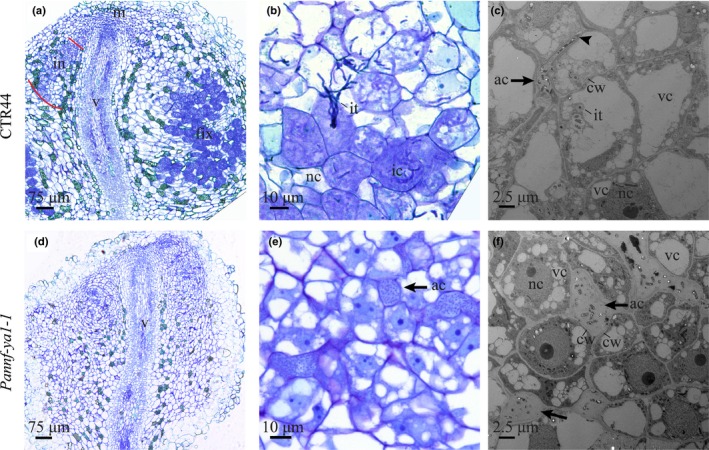
*PanNF‐YA1* is essential for intracellular rhizobium infection. (a, b) Nodule cytoarchitecture of *Parasponia andersonii* transgenic control (CTR44) plants studied by light microscopy. (a) Sections of a mature transgenic control nodule. The infection zone (in) in one lobe is marked with red lines. (b) Formation of intracellular infection threads. Shown is a close‐up of the infection zone of a mature nodule. (c) Transmission electron microscopy image of apoplastic rhizobium infection (arrow) and initiation of intracellular infection (arrowhead) in a transgenic control nodule. (d, e) Cytoarchitecture of a Pannf‐ya1 nodule studied by light microscopy. *Pannf‐ya1* mutant nodules lack intracellular infection threads (d). In mature *Pannf‐ya1‐1* nodules (e), apoplastic colonies of rhizobium can be detected (arrow). (f) Transmission electron microscopy image of large apoplastic rhizobium colonies (arrows) in a *Pannf‐ya1* mutant nodule. Plastic sections (a, b, d, e) were stained using Toluidine Blue. m, nodule meristem; in, infection zone; fix, fixation zone; v, nodule vasculature; it, intracellular infection thread; ic, infected cells; nc, noninfected cells; ac, apoplastic colonies of rhizobia; cw, cell wall; nc, nucleus; vc, vacuoles. Nodules were isolated at 4 wk post‐inoculation with *Mesorhizobium plurifarium* BOR2.

Next, we studied the cytoarchitecture of *Pannf‐ya1‐1* nodules using light microscopy as well as transmission electron microscopy. In wild‐type *Parasponia*, rhizobium bacteria first colonize the apoplast of the nodule infection zone, after which they enter nearby cells through infection threads (Lancelle & Torrey, 1984; Fig. 3a,b). *Parasponia andersonii nf‐ya1‐1* mutant nodules display a wild‐type cytology, but cells in the infection zone are devoid of intracellular infection threads (Fig. 3d,e). Instead, large apoplastic colonies of rhizobium can be seen that occasionally occupy dead host cells (Fig. 3e). Transmission electron microscopy showed that apoplastic rhizobia in wild‐type nodules are embedded in a thin layer of secreted matrix material from where intracellular infection can occur (Fig. 3c; Trinick, 1979). By contrast, no such intracellular infections were observed in *Pannf‐ya1‐1* mutant nodules. Instead, rhizobium formed large apoplastic colonies embedded in a secreted matrix (Fig. 3f). This infection phenotype was confirmed in two additional *Pannf‐ya1* mutant lines (Figs S4c,d, S6c,d). Based on these results, we conclude that *PanNF‐YA1* has an essential role in intracellular infection thread formation in *Parasponia* nodules.

### PanNF‐YA3 and PanNF‐YA6 are expressed during nodule formation

The *nf‐ya1* mutants in *M. truncatula* and *L. japonicus* are clearly affected in nodule development (Combier *et al*., 2006; Soyano *et al*., 2013; Laporte *et al*., 2014; Xiao *et al*., 2014). By contrast, no such phenotype was observed in *P. andersonii nf‐ya1‐1* mutants. Therefore, we questioned whether additional *NF‐YA*‐encoding genes perform a symbiotic function in *Parasponia*.

**Figure 4:**
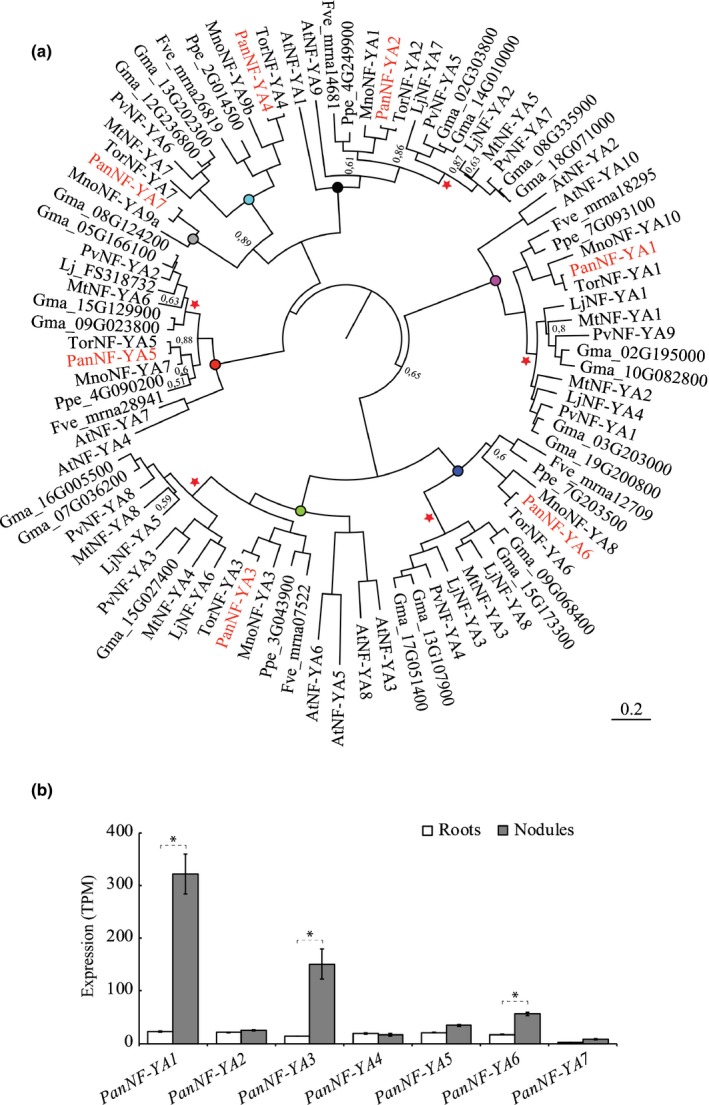
Phylogenetic relation and symbiotic expression of *Parasponia andersonii NF‐YA* genes. (a) Bayesian phylogeny of NF‐YA proteins reconstructed based on an alignment of protein sequences from the following species: *Parasponia andersonii* (Pan), *Trema orientalis* (Tor), *Arabidopsis thaliana* (At), *Medicago truncatula* (Mt), *Lotus japonicus* (Lj), *Glycine max* (Gma), *Phaseolus vulgaris* (Pv), *Morus notabilis* (Mno), *Prunus persica* (Ppe), *Fragaria vesca* (Fve). *Parasponia andersonii* NF‐YA proteins are marked in red. Red pentagrams mark duplication events within the legume family. Orthogroups are indicated by a coloured circle. Node labels indicate posterior probability, Node labels with a value > 0.9 are not shown. (b) Expression level of *PanNF‐YA* genes in roots and mature nodules. Expression was determined by quantification of RNAseq reads. Data represent average expression in transcripts per million (TPM) (*n* = 3) ± SD, which were obtained from van Velzen *et al*. (2018). Nodules were isolated 4 wk post‐inoculation with *Mesorhizobium plurifarium* BOR2. *, *P* < 0.01 adjusted for multiple testing based on false discovery rate estimated for two‐fold change in mature nodule vs root sample as described by van Velzen *et al*. (2018).

To determine whether close paralogs of *PanNF‐YA1* exist in *P. andersonii*, as has been reported for the model legumes *M. truncatula* and *L. japonicus* (Laloum *et al*., 2013; Soyano *et al*., 2013), we reconstructed the phylogeny of the NF‐YA clade. This revealed that *P. andersonii* possesses seven *NF‐YA* genes that are divided over seven orthogroups (Figs 4a, S7). We noted that legumes experienced gene duplication events in five orthogroups, including the *NF‐YA1* lineage (Fig. 4a). In line with this, we conclude that *PanNF‐YA1* is the sole orthologue of two legume genes represented by *MtNF‐YA1*/*LjNF‐YA1* and *MtNF‐YA2*/*LjNF‐YA4* in *M. truncatula* and *L. japonicus*. Additionally, we noted that legumes have genes only in six orthogroups, lacking an orthologue of *PanNF‐YA7*. To determine whether gene duplications are specific to legumes, we reconstructed the phylogeny also including the *NF‐YA* protein family of the actinorhizal plant species *Casuarina glauca* (Fagales) and *Datisca glomerata* (Cucurbitales)*,* and the legume *Arachis duranensis*. This showed that *C. glauca* and *D. glomerata* generally possess a single gene in each of the seven orthogroups, similar to what was observed for *P. andersonii*, supporting the conclusion that duplication of *NF‐YA* genes in legumes is the result of a lineage‐specific event (Fig. S7).

To study whether other *PanNF‐YA* genes might function in rhizobium symbiosis, we determined their expression in nodules using published transcriptome data (van Velzen *et al*., 2018). This revealed that six *PanNF‐YA* genes are expressed in nodules (transcripts per million > 10), three of which show a nodule‐enhanced expression profile, namely *PanNF‐YA1, PanNF‐YA3* and *PanNF‐YA6*, respectively (Fig. 4b). To study the symbiotic expression of *PanNF‐YA3* and *PanNF‐YA6* in more detail, we created promoter‐reporter GUS constructs for both genes. These constructs contain 3.5 and 4.9 kb upstream of the translational start sites of *PanNF‐YA3* and *PanNF‐YA6*, respectively.

**Figure 5:**
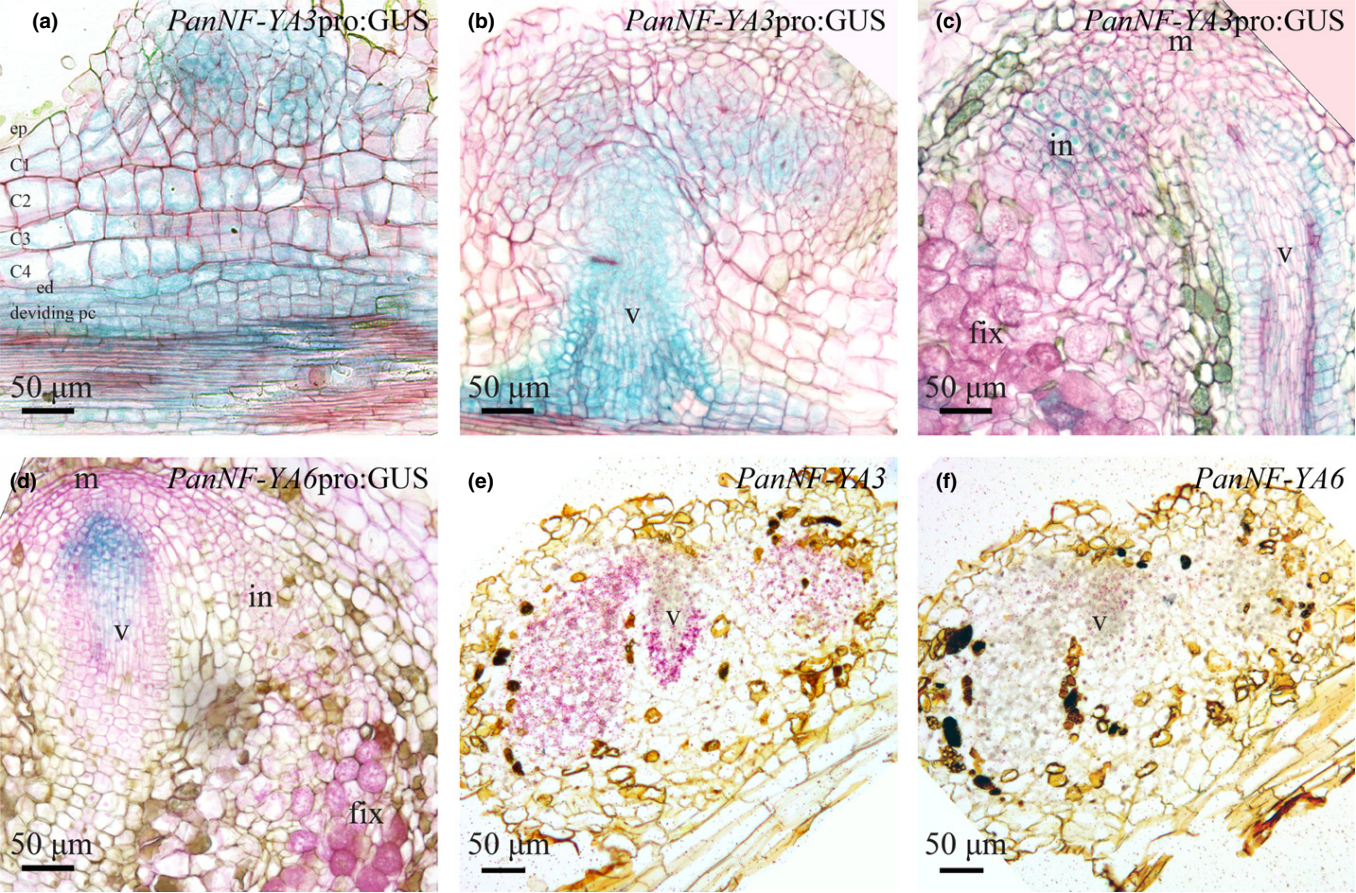
Spatiotemporal expression pattern of *PanNF‐YA3* and *PanNF‐YA6* in *Parasponia andersonii* root nodules. (a, c) Spatiotemporal expression pattern of *PanNF‐YA3*pro:GUS in nodules of different developmental stages. (a) *PanNF‐YA3*pro:GUS activity is observed in dividing epidermal, cortical, endodermal cells of a nodule primordium as well as the root vasculature. (b) In a young nodule, *PanNF‐YA3*pro:GUS activity is confined to the nodule lobes that will become intracellularly infected and the nodule vasculature. (c) In a mature nodule *PanNF‐YA3*pro:GUS is active in the infection zone and the nodule vasculature (v). (d) *PanNFYA6*pro:GUS is active at the nodule vascular meristem. (e, f) Spatiotemporal expression pattern of *PanNFYA3* and *PanNF‐YA6* visualized by *in situ* hybridization on consecutive sections of a young *P. andersonii* nodule primordium. ep, epidermis; C1–C4, first to fourth cortical cell layer; ed, endodermis; pc, pericycle; m, nodule meristem; in, infection zone; fix, fixation zone; v, nodule vasculature. In (a)–(d), sections (7 µm) were counterstained with Ruthenium Red. Nodules were isolated at 4 wk post‐inoculation with *Mesorhizobium plurifarium* BOR2.

Transgenic *P. andersonii* lines harbouring these constructs revealed that the *PanNF‐YA3_pro_:GUS* construct is active in the root apical meristem (Fig. S8a). In the case of *PanNF‐YA6*, the promoter‐reporter construct is expressed in young parts of the roots, including the meristem (Fig. S8e). Next, we studied their expression patterns following inoculation with rhizobium. In nodule primordia, *PanNF‐YA3_pro_*:*GUS* is active in the dividing epidermal, cortical and pericycle cells, mimicking activity of the *PanNF‐YA1_pro_*:*GUS* reporter (Figs 5a, S8b,c). In young nodules, *PanNF‐YA3_pro_*:*GUS* is expressed in the central region of the nodule lobes where rhizobium infection occurs and in the vascular bundle (Fig. 5b). In mature nodules, *PanNF‐YA3_pro_*:*GUS* activity is observed in the infection zone and nodule vasculature (Figs 5c, S8d). Activity of the *PanNF‐YA6_pro_*:*GUS* reporter is restricted to the nodule vascular meristem (Figs 5d, S8f). ISH confirmed the expression patterns of *PanNF‐YA3* and *PanNF‐YA6* in young nodules (Fig. 5e,f). Additionally, it showed that *PanNF‐YA3* is coexpressed with *PanNIN* in the lobes of young nodules (Figs 1f, 5e). Therefore, we questioned whether symbiotic *PanNF‐YA3* and/or *PanNF‐YA6* expression requires a functional *PanNIN* gene. qRT‐PCR experiments on the same samples used for studying *PanNF‐YA1* expression revealed that neither *PanNF*‐*YA3* nor *PanNF‐YA6* expression is enhanced within 24 h after inoculation (Fig. S8g,h).

Taken together, these data suggest a possible symbiotic role for *PanNF‐YA3* and, to a lesser extent, *PanNF‐YA6,* although in roots both genes are not responsive to rhizobium inoculation (1 dpi)*.*

### *PanNF‐YA1, PanNF‐YA3* and *PanNF‐YA6* act redundantly in nodule development

To determine the role of *PanNF‐YA3* and *PanNF‐YA6* during *Parasponia* nodule formation, we created CRISPR/Cas9 mutants for both genes. *Pannf‐ya3* and *Pannf‐ya6* knockout mutant lines were created using three sgRNAs targeting the second and third exons, respectively (Fig. S9a–d). Inoculation with *M. plurifarium* BOR2 showed that *Pannf‐ya3* and *Pannf‐ya6* mutants developed a similar number of nodules as transgenic control plants (Fig. S9e). These mutant nodules were able to fix N_2_, as determined by ARA (Fig. S9f), and display a wild‐type cytoarchitecture (Fig. S9g,h). This indicates that neither *PanNF‐YA3* nor *PanNF‐YA6* is essential for *Parasponia* nodule formation.

As we cannot rule out the possibility that *PanNF‐YA1, PanNF‐YA3* and/or *PanNF‐YA6* function redundantly in nodule organogenesis, we decided to create three double mutants (*Pannf‐ya1;Pannf‐ya3*, *Pannf‐ya1;Pannf‐ya6* and *Pannf‐ya3;Pannf‐ya6*)*,* and higher‐order triple mutants (*Pannf‐ya1;Pannf‐ya3;Pannf‐ya6*; Fig. S10). When inoculated with *M. plurifarium* BOR2, all three double mutant combinations formed nodules (Fig. S11a). Consistent with the phenotype of *Pannf‐ya1* single mutant nodules, *Pannf‐ya1;Pannf‐ya3‐1* and *Pannf‐ya1;Pannf‐ya6‐6* double mutant nodules are devoid of intracellular infection structures (Fig. S12a,b,d,e). Intracellular infection in *Pannf‐ya3;Pannf‐ya6‐5* double mutant nodules was not affected (Fig. S12c,f), indicating that intracellular rhizobium infection of *P. andersonii* nodules is specifically controlled by *PanNF‐YA1*.

**Figure 6:**
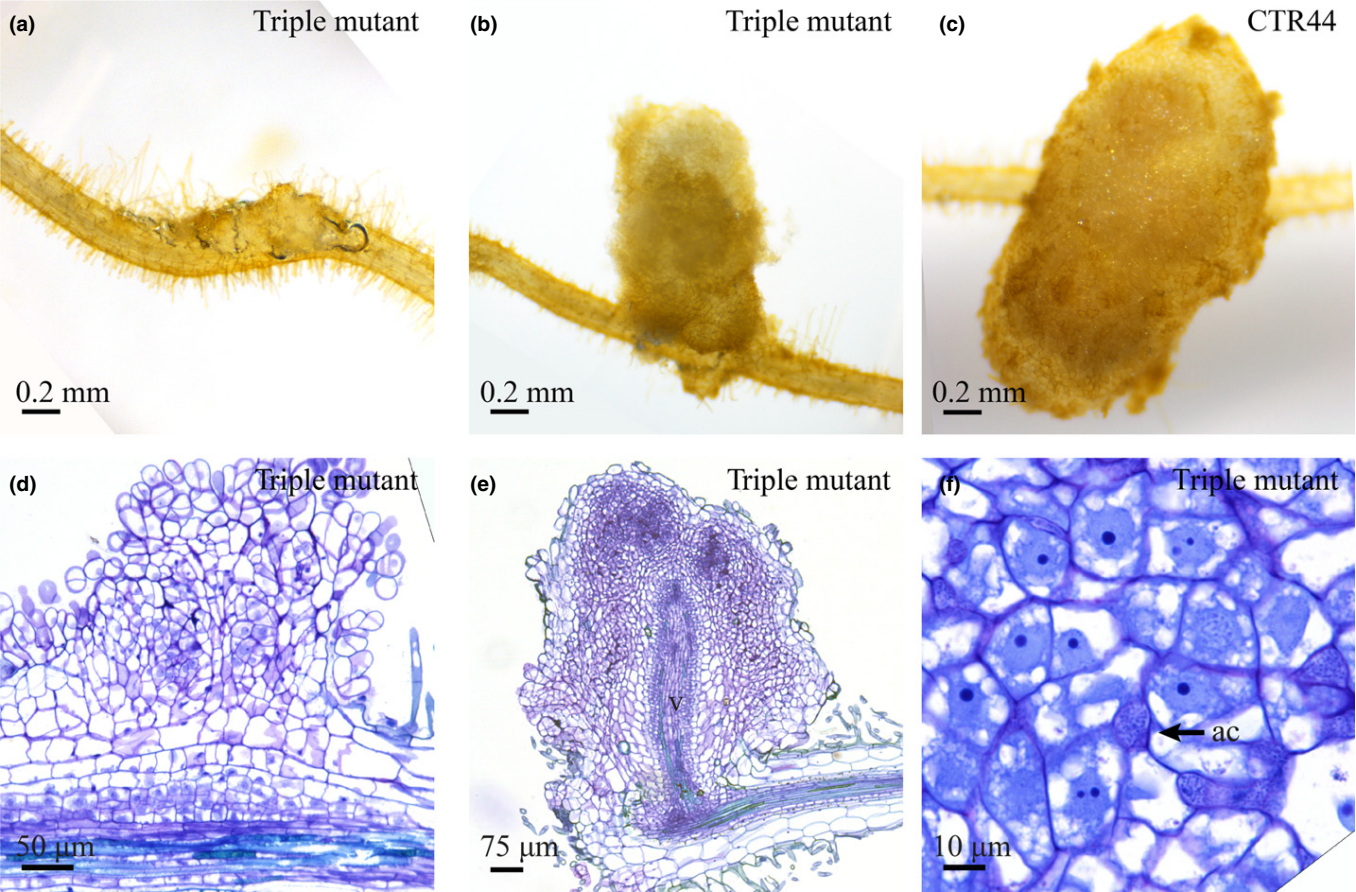
The *Pannf‐ya1;Pannf‐ya3;Pannf‐ya6* triple mutant is affected in nodule development. (a, b). Nodule‐like structures formed on a *Pannf‐ya1;Pannf‐ya3;Pannf‐ya6* mutant. (c) Nodule formed on a transgenic control line (CTR44). (d, e) Sections of the nodule‐like structure shown in (a) and (b). (f) Apoplastic rhizobia (arrow) in a *Pannf‐ya1;Pannf‐ya3;Pannf‐ya6* mutant nodule, whereas intracellular infection is absent. v, nodule vasculature; ac, apoplastic colonies of rhizobia. Sections were stained using Toluidine Blue. Nodules were isolated at 4 wk post‐inoculation with *Mesorhizobium plurifarium* BOR2.

Next, we analysed the nodulation phenotype of three independent *Pannf‐ya1;Pannf‐ya3;Pannf‐ya6* triple mutant lines. All three lines showed initiation of nodule organogenesis upon rhizobium inoculation with similar efficiency when compared to the transgenic control (Fig. S11a). However, *Pannf‐ya1;Pannf‐ya3;Pannf‐ya6* triple mutants nodules were irregular in shape and remain substantially smaller than nodules formed on the control (Figs 6a–c, S11b). Approximately half of the *Pannf‐ya1;Pannf‐ya3;Pannf‐ya6* triple mutant nodules do not develop beyond the primordial stage (Fig. 6a). These nodule‐like structures originated from multiple rounds of cell divisions in the epidermis and outer cortex, but did not develop a vascular bundle (Fig. 6d). By contrast, the somewhat larger nodules formed on the *Pannf‐ya1;Pannf‐ya3;Pannf‐ya6* triple mutant developed a nodule vascular bundle, but were disturbed in growth (Figs 6b,e, S11b). In *M. truncatula*, it was shown that the casparian strip was absent from the nodule endodermis in the region close to the meristem (Xiao *et al*., 2014). We used this criterion to determine whether or not the nodule meristem of the *P. andersonii Pannf‐ya1;Pannf‐ya3;Pannf‐ya6* triple mutants remained active. Nodule sections were examined under UV light to detect light emitted by the casparian strips. This showed that the meristematic region in *P. andersonii* triple mutant nodules is fully surrounded by casparian strips, which was not observed in wild‐type nodules of a similar age (Fig. S13). This result indicates that meristematic activity ceased early in the development of *Pannf‐ya1;Pannf‐ya3;Pannf‐ya6* triple mutant nodules. Like *Pannf‐ya1* single mutant nodules, *Pannf‐ya1;Pannf‐ya3;Pannf‐ya6* triple mutant nodules contain large apoplastic colonies of rhizobium, but are devoid of intracellular infection structures (Fig. 6f). Taken together, these data demonstrate that rhizobium intracellular infection is specifically controlled by *PanNF‐YA1*, and that *PanNF‐YA1*, *PanNF‐YA3* and *PanNF‐YA6* function redundantly to control nodule growth and development.

As *P. andersonii nf‐ya1* mutant nodules are devoid of intracellular infection, we questioned whether this is specific for rhizobium or, alternatively, whether *NF‐YA* genes may also function in intracellular colonization by arbuscular mycorrhizal fungi. To test this, control plants, the *Pannf‐ya1*, *Pannf‐ya3* and *Pannf‐ya6* single mutants, and the *Pannf‐ya1;Pannf‐ya3;Pannf‐ya6* triple mutant were grown under phosphate‐poor conditions and inoculated with 250 spores of the *Rhizophagus irregularis* strain DOAM197198. The average colonization and arbuscule formation frequency were scored at 6 wk post‐inoculation. This showed that all mutants were equally well mycorrhized when compared with control plants (Fig. S14). Therefore, we conclude that *PanNF‐YA1* has a specific role in rhizobium intracellular infection.

## Discussion

The transcription factors *NIN* and *NF‐YA1* are essential components in a transcriptional network controlling rhizobium‐induced nodule formation in legumes (Soyano & Hayashi, 2014). Here, we showed that the orthologous genes – *PanNIN* and *PanNF‐YA1* – are essential for the formation of functional root nodules in the nonlegume *P. andersonii*. Earlier studies, using transient RNA interference‐mediated knockdown, indicated that *CgNIN* also has a symbiotic function in the nodulating actinorhizal species *Casuarina glauca* (Clavijo *et al*., 2015). The *Parasponia* (Rosales), *Casuarina* (Fagales) and legume (Fabales) lineages diverged *c*. 110 Ma, soon after an assumed shared evolutionary event that gave birth to the nodulation trait (Soltis *et al*., 1995; Wang *et al*., 2009; van Velzen *et al*., 2019). As *NIN* and *NF‐YA1* are indispensable for the formation of functional N_2_‐fixing nodules in distinct taxonomic lineages, we conclude that these transcription factors represent core genes in the nodulation trait. Furthermore, we hypothesize that this recruitment into the nodulation trait has occurred in a species ancestral to the Fabales, Fagales, Cucurbitales and Rosales split.

In *L. japonicus*, *LjNF‐YA1* is a direct transcriptional target of LjNIN (Soyano *et al*., 2013, 2015). Direct evidence of a similar relationship has not been provided in any other species. Experiments presented here showed that in *P. andersonii*, rhizobium‐induced *PanNF‐YA1* expression is PanNIN‐dependent and that both genes are coexpressed in nodule primordia. In line with the hypothesis that both genes have been recruited in nodulation in a common ancestor of legumes and *Parasponia*, it is likely that the direct transcriptional regulation of the *NF‐YA1* gene by NIN is conserved in nodulating species. This hypothesis is supported by the occurrence of putative NIN‐binding sites in the promoter region of *PanNF‐YA1* (Fig. S15). In case these bindings sites find experimental support, the question remains whether the NIN‐NF‐YA1 transcription factor module is ancestral to the N_2_‐fixing clade, or whether it has evolved in concurrence with the nodulation trait.

*Parasponia andersonii NF‐YA1* controls intracellular rhizobium infection, and knockout mutants of this gene are specifically blocked in infection thread formation. This mutant phenotype is different from the phenotypes reported for legume *nf‐ya1* knockout and/or knockdown lines. In *L. japonicus* and *M. truncatula*, *nf‐ya1* mutants and RNAi knockdown lines form smaller nodules (Combier *et al*., 2006; Soyano *et al*., 2013; Laporte *et al*., 2014; Hossain *et al*., 2016). In *M. truncatula*, this developmental phenotype is a result of absence or reduced activity of the nodule meristem (Xiao *et al*., 2014), whereas in *L. japonicus LjNF‐YA1* is indispensable for nodule differentiation, including vascular bundle formation (Hossain *et al*., 2016). Absence of a functional *Mtnf‐ya1* gene in *M. truncatula* also affects rhizobium infection, resulting in an increased number of infection threads that are arrested in the epidermis, and often have a swollen, more bulbous appearance (Laloum *et al*., 2014; Laporte *et al*., 2014). In *P. andersonii nf‐ya1* knockout mutants are not affected in nodule development. This divergence in phenotype between *P. andersonii* and legumes is most probably the result of adaptive evolution and subsequent divergence of the nodulation trait in both lineages. For example, intracellular rhizobium infection in *M. truncatula* and *L. japonicus* is initiated in curled root hairs, whereas in *P. andersonii* only nodule cells become invaded. Consequently, infection phenotypes may be observed in different cell types.

Papilionoideae legumes (e.g. *L. japonicus*, *M. truncatula,* soybean (*Glycine max*), and common bean (*Phaseolus vulgaris*)) experienced gene duplication events in five *NF‐YA* orthogroups, including *NF‐YA1*, which is most probably the result of whole‐genome duplication in a common ancestor (Cannon *et al*., 2006; Young *et al*., 2011). Subsequent gene redundancy may have allowed subneofunctionalization of *NF‐YA1* and its closest paralogue in legumes. Phenotypic analyses of mutant plants where both *NF‐YA1* paralogues are targeted simultaneously support the idea that controlling rhizobium intracellular infection is an ancestral symbiotic function of NF‐YA1 and its closest paralogue. For example, by committing *MtNF‐YA2* RNAi in a *M. truncatula nf‐ya1* mutant background, a more severe rhizobium infection phenotype can be observed (Laloum *et al*., 2014). Also, in common bean, a strong infection phenotype is observed after silencing of both *PvNF‐YA9* and *PvNF‐YA1* in *A. rhizogenes*‐transformed roots (Rípodas *et al*., 2019). However, in this study, single gene targets have not been analysed. Such gene duplications, which are common in Papilionoideae legumes, complicate reverse genetic studies. *P. andersonii* did not experience any duplication events in any of the seven *NF‐YA* orthogroups (Fig. 4a). In line with this, we argue that this species may be more suited to uncover the functioning of *NF‐YA* genes by reverse genetics.

We also studied the function of two additional *NF‐YA* genes (*PanNF‐YA3* and *PanNF‐YA6*) in *P. andersonii*, as both these genes have a nodule‐enhanced expression profile (Fig. 4b). Such a nodule‐enhanced expression profile has also been reported for the *M. truncatula* orthologues *MtNF‐YA8* (orthologous to *PanNF‐YA3*) and *MtNF‐YA3* (orthologous to *PanNF‐YA6*; Baudin *et al*., 2015). However, no apparent nodulation phenotype could be observed in *P. andersonii* single and double mutants. Only upon creating a higher‐order *Pannf‐ya1;Pannf‐ya3;Pannf‐ya6* mutant was an effect on nodule organogenesis observed. This suggests that all three *PanNF‐YA* genes act redundantly in controlling nodule development.

Recent phylogenomic analyses revealed that, within the N_2_‐fixing clade, absence of the nodulation trait is associated with pseudogenization of the *NIN* gene (Griesmann *et al*., 2018; van Velzen *et al*., 2018). This shows that within the N_2_‐fixing clade the functioning of this gene correlates with the nodulation trait. In contrast to *NIN*, no such correlation has been reported between the presence of *NF‐YA1* orthologues and the nodulation trait (Griesmann *et al*., 2018; van Velzen *et al*., 2018), suggesting that these genes also have nonsymbiotic functions. *Arabidopsis thaliana* has two orthologues of *LjNF‐YA1, MtNF‐YA1* and *PanNF‐YA1*, named *AtNF‐YA2* and *AtNF‐YA10* (Fig. 4a). Mutant analysis of these genes has been hampered by the sterility phenotype of *Atnf‐ya2* insertion and RNAi lines (Pagnussat *et al*., 2005; Sorin *et al*., 2014). Misexpression studies of either gene revealed a function in leaf and root growth and lateral root initiation as well as increased tolerance to several types of abiotic stresses (Leyva‐González *et al*., 2012; Sorin *et al*., 2014; Zhang *et al*., 2017; Soyano *et al*., 2019). Furthermore, it was shown that, in *L. japonicus*, ectopic expression of *LjNF‐YA1* results in lateral roots with malformed tips (Soyano *et al*., 2013; Sorin *et al*., 2014) We observed a mild, though consistent, decrease in lateral roots formed in plantlets containing a mutation in *Pannf‐ya1*. This supports the findings that *NF‐YA1* orthologous genes have a nonsymbiotic function in root development, and may explain why *NF‐YA1* is not pseudogenized in species that have lost the nodulation trait (Soyano *et al*., 2013; Griesmann *et al*., 2018; van Velzen *et al*., 2018). As the *P. andersonii nf‐ya1* knockout mutants are not affected in the symbiosis with arbuscular mycorrhiza, it suggests that NF‐YA1 symbiotic functioning is exclusively required to allow entry of symbiotic bacteria. As the bacterial infectability of cells is a key characteristic of the nodulation trait, it will be an important future scientific objective to determine the core transcriptional network regulated by *NF‐YA1* and its interacting partners. Having a *P. andersonii nf‐ya1* mutant available with a strict infection phenotype as a comparative system to legumes where infection and organogenesis phenotypes are intertwined will be instrumental to achieving this objective.

## Author contributions

FB, LR and RG planned and designed the research; FB, LR, MR‐F, OK and YPR performed the experiments; FB, AvZ, LR, RG, TB, TO and YPR analysed the data; and FB, AvZ and RG wrote the manuscript.

## Supporting information

Please note: Wiley‐Blackwell are not responsible for the content or functionality of any supporting information supplied by the authors. Any queries (other than missing material) should be directed to the *New Phytologist* Central Office.

**Fig. S1** Spatiotemporal expression pattern of PanNF‐YA1pro:GUS in *Parasponia andersonii* roots.
**Fig. S2** Structure and expression of the *P. andersonii NIN* gene and the genotype of CRISPR‐Cas9 *Pannin* mutants.
**Fig. S3** *Rhizobium tropici* CIAT899.pMP604 constitutively expresses the LCO biosynthesis gene *nodC.*
**Fig. S4** Structure of the *P. andersonii NF‐YA1* gene and genotype of CRISPR‐Cas9 *Panf‐ya1* mutants.
**Fig. S5** Lateral root formation is affected in the *P. andersonii nf‐ya1* mutant.
**Fig. S6** Phenotyping of *P. andersonii nf‐ya1* knockout mutants.
**Fig. S7** Phylogenetic analysis of NF‐YA in the nitrogen‐fixing clade.
**Fig. S8** Expression of *PanNF‐YA3* and *PanNF‐YA6* in *P. andersonii* roots and nodules.
**Fig. S9** Gene structure of *P. andersonii NF‐YA3* and *NF‐YA6,* genotype of CRISPR‐Cas9 mutants, and nodulation phenotypes.
**Fig. S10** Genotypes of *Pannf‐ya1, Pannf‐ya3* and *Pannf‐ya6* CRISPR‐Cas9 double and triple mutants.
**Fig. S11** Nodulation efficiency and nodule size of *Parasponia andersonii nf‐ya* single, double and triple knockout mutants.
**Fig. S12** Nodule cytoarchitecture of *Parasponia andersonii nf‐ya* double knockout mutants.
**Fig. S13** Casparian strips in the vascular endodermis next to the nodule meristem in *Pannf‐ya1;Pannf‐ya3;Pannf‐ya6* mutant plants.
**Fig. S14** *Parasponia andersonii nf‐ya1, nf‐ya3, nf‐ya6* and *Pannf‐ya1;Pannf‐ya3;Pannf‐ya6* mutants can form arbuscular mycorrhiza. *Parasponia andersonii* nf‐ya1, nf‐ya3 and nf‐ya6 knockout mutants can form arbuscular mycorrhiza.
**Fig. S15** Putative NIN‐binding sites in the PanNF‐YA1 promoter region.
**Table S1** Sequences of sgRNAs used for creating single, double and triple knockout mutants.
**Table S2** Primers used in this work.
**Table S3** Putative promoter sequences used for promoter‐reporter GUS assays.
**Table S4** Gene identifiers for NF‐YA proteins used to build the phylogenetic tree depicted in Figs 4, S7.
