# Supplementary material for: Mutant analysis in the nonlegume Parasponia andersonii identifies NIN and NF‐YA1 transcription factors as a core genetic network in nitrogen‐fixing nodule symbioses
Source: New Phytol. 2020 Jan 30;226(2):541–54. doi: 10.1111/nph.16386 (PMC7154530; doi:10.1111/nph.16386)
Supplement: Supplementary file 1 — Fig. S1 Spatiotemporal expression pattern of PanNF‐YA1pro:GUS in Parasponia andersonii roots. Fig. S2 Structure and expression of the P. andersonii NIN gene and the genotype of CRISPR‐Cas9 Pannin mutants. Fig. S3 Rhizobium tropici CIAT899.pMP604 constitutively expresses the LCO biosynthesis gene nodC. Fig. S4 Structure of the P. andersonii NF‐YA1 gene and genotype of CRISPR‐Cas9 Panf‐ya1 mutants. Fig. S5 Lateral root formation is affected in the P. andersonii nf‐ya1 mutant. Fig. S6 Phenotyping of P. andersonii nf‐ya1 knockout mutants. Fig. S7 Phylogenetic analysis of NF‐YA in the nitrogen‐fixing clade. Fig. S8 Expression of PanNF‐YA3 and PanNF‐YA6 in P. andersonii roots and nodules. Fig. S9 Gene structure of P. andersonii NF‐YA3 and NF‐YA6, genotype of CRISPR‐Cas9 mutants, and nodulation phenotypes. Fig. S10 Genotypes of Pannf‐ya1, Pannf‐ya3 and Pannf‐ya6 CRISPR‐Cas9 double and triple mutants. Fig. S11 Nodulation efficiency and nodule size of Parasponia andersonii nf‐ya single, double and triple knockout mutants. Fig. S12 Nodule cytoarchitecture of Parasponia andersonii nf‐ya double knockout mutants. Fig. S13 Casparian strips in the vascular endodermis next to the nodule meristem in Pannf‐ya1;Pannf‐ya3;Pannf‐ya6 mutant plants. Fig. S14 Parasponia andersonii nf‐ya1, nf‐ya3, nf‐ya6 and Pannf‐ya1;Pannf‐ya3;Pannf‐ya6 mutants can form arbuscular mycorrhiza. Parasponia andersonii nf‐ya1, nf‐ya3 and nf‐ya6 knockout mutants can form arbuscular mycorrhiza. Fig. S15 Putative NIN‐binding sites in the PanNF‐YA1 promoter region. Table S1 Sequences of sgRNAs used for creating single, double and triple knockout mutants. Table S2 Primers used in this work. Table S3 Putative promoter sequences used for promoter‐reporter GUS assays. Table S4 Gene identifiers for NF‐YA proteins used to build the phylogenetic tree depicted in Figs 4, S7. [file NPH-226-541-s001.pdf]

New Phytologist **Supporting Information**

Article title:

Mutant analysis in the non-legume *Parasponia andersonii* identifies NIN and NF-YA1 transcription factors as a core genetic network in nitrogen-fixing nodule symbioses

Authors:
Fengjiao Bu, Luuk Rutten, Yuda Purwana Roswanjaya, Olga Kulikova, Marta Rodriguez-Franco, Thomas Ott, Ton Bisseling, Arjan van Zeijl & Rene Geurts

Article acceptance date: 4 December 2019

The following Supporting Information is available for this article:

Fig. S1: Spatiotemporal expression pattern of *PanNF-YA1*$_{pro}$:GUS in *Parasponia andersonii* roots.

Fig. S2: Structure and expression of the *P. andersonii NIN* gene and the genotype of CRISPR-Cas9 *Pannin* mutants.

Fig. S3: *Rhizobium tropici* CIAT899.pMP604 constitutively expresses the LCO biosynthesis gene *nodC.*

Fig. S4: Structure of the *P. andersonii NF-YA1* gene and genotype of CRISPR-Cas9 *Panf-ya1* mutants.

Fig. S5: Lateral root formation is affected in the *P. andersonii nf-ya1* mutant.

Fig. S6: Phenotyping of *P. andersonii nf-ya1* knockout mutants.

Fig. S7: Phylogenetic analysis of NF-YA in the nitrogen-fixing clade.

Fig. S8: Expression of *PanNF-YA3* and *PanNF-YA6* in *P. andersonii* roots and nodules.

Fig. S9: Gene structure of *P. andersonii NF-YA3* and *NF-YA6*, genotype of CRISPR-Cas9 mutants, and nodulation phenotypes.

Fig. S10: Genotypes of *Pannf-ya1*, *Pannf-ya3* and *Pannf-ya6* CRISPR-Cas9 double and triple mutants.

Fig. S11: Nodulation efficiency and nodule size of *Parasponia andersonii nf-ya* single, double and triple knockout mutants.

Fig. S12: Nodule cytoarchitecture of *Parasponia andersonii nf-ya* double knockout mutants.

Fig. S13: Casparian strips in the vascular endodermis next to the nodule meristem in *Pannf-ya1;Pannf-ya3;Pannf-ya6* mutant plants.

Fig. S14: *Parasponia andersonii nf-ya1, nf-ya3, nf-ya6* and *Pannf-ya1;Pannf-ya3;Pannf-ya6* mutants can form arbuscular mycorrhiza.

Fig. S15: Putative NIN Binding sites in the PanNF-YA1 promoter region.

Table S1 Sequences of sgRNAs used for creating single, double and triple knockout mutants.

Table S2 Primers used in this work.

Table S3 Putative promoter sequences used for promoter-reporter GUS assays.

Table S4 Gene identifiers for NF-YA proteins used to build the phylogenetic tree depicted in Figure 4 and Figure S7.

Fig. S1: Spatiotemporal expression pattern of *PanNF-YA1*$_{pro}$:GUS in *Parasponia andersonii* roots.

(**a-d**) GUS-stained non-inoculated root segments. (e-g) GUS-stained inoculated root segments. (**a**) Faint *PanNF-YA1*$_{pro}$:GUS activity was observed around the vasculature in the differentiated zone of a young root. (**b**) *PanNF-YA1*$_{pro}$:GUS activity observed in the pericycle cells (70 µm-thick longitudinal root section). (**c**) *PanNF-YA1*$_{pro}$:GUS activity observed in pericycle cells opposite protoxylem poles (70 µm-thick cross-section of the root). (**d**) Spatiotemporal expression of *PanNF-YA1*$_{pro}$:GUS during lateral root initiation (70 µm-thick longitudinal root-section). (**e, f**) *PanNF-YA1*$_{pro}$:GUS activity was induced in epidermal cells at the elongation and differentiation zone of a root at 2 dpi with *M. plurifarium* strain BOR2. (**g**) *PanNF-YA1*$_{pro}$:GUS activity detected in the root epidermis upon rhizobium inoculation at a similar developing stage as shown in Figure 1A. Plants were grown *in vitro* (a-f), or in a perlite potting system (**g**). Data shown are obtained using transgenic *PanNF-YA1*$_{pro}$:GUS line 1E5. ep: epidermis; pc: pericycle; px: protoxylem; arrowhead indicates lateral root primordia.

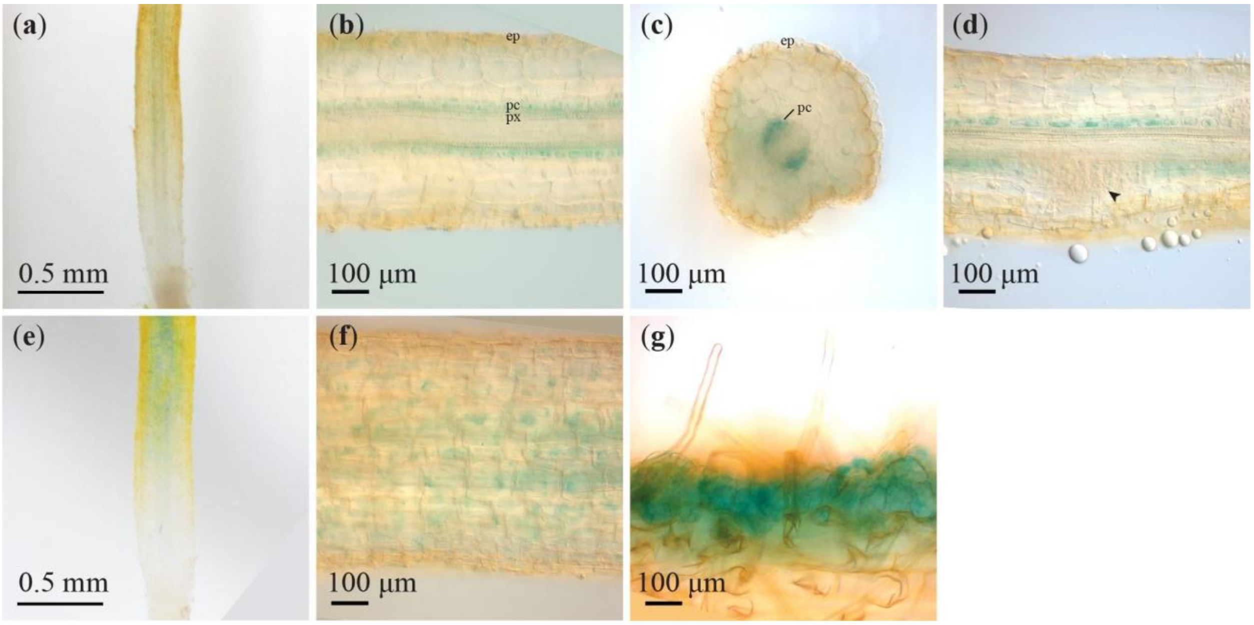

Fig. S2: Structure and expression of the *P. andersonii NIN* gene and the genotype of CRISPR-Cas9 *Pannin* mutants..

(**a**) Schematic representation of the *PanNIN* gene model. Indicated are two *PanNIN* transcripts: *PanNIN.1* and *PanNIN.2*. Translational initiation (ATG) and termination (STOP) sites are indicated by arrows. (**b**) Expression of *PanNIN.1* and *PanNIN.2* in roots and nodules. Expression was determined by quantification of RNAseq reads. Data represent DE-seq2-normalized read counts ($n = 3$) ± SD, which were obtained from van Velzen et al., 2018. Different letters indicate statistical significance (Student's t-test, $p < 0.05$). (**c**) Schematic representation of the *PanNIN* gene model. Indicated are the locations of 3 sgRNA target sites (red triangles) and the translational initiation sites present in *PanNIN.1* and *PanNIN.2*. Note that the sgRNAs target the first coding-exon that is specific for *PanNIN.1*. (**d**) Alignment of the sequence of the first *PanNIN* coding-exon in wild type and *Pannin* mutant lines B1 and B3. sgRNA target sites are marked in blue, PAM sequences are marked in red. Note that in both lines mutations are homozygous.

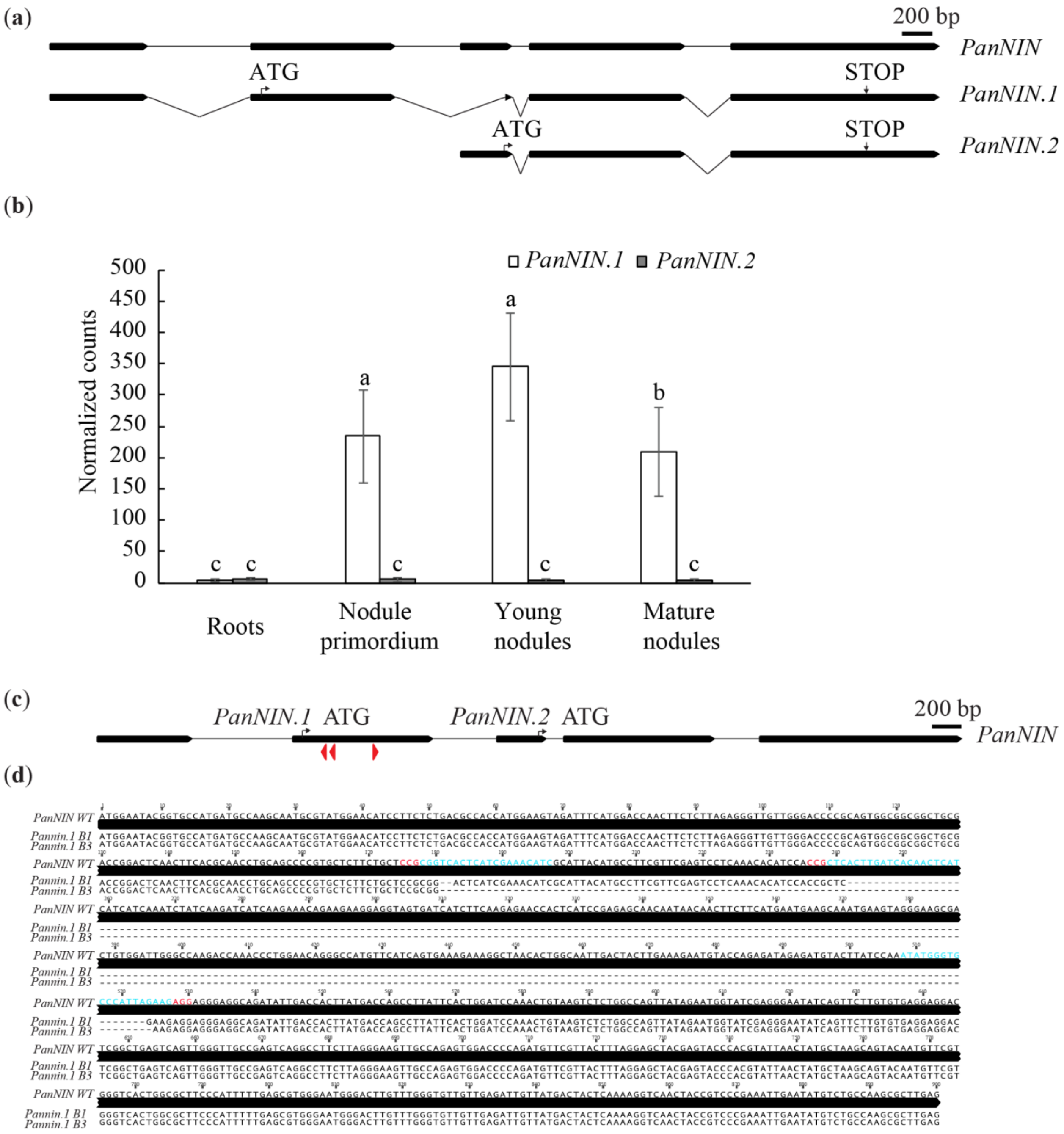
(a)
200 bp
PanNIN
ATG
STOP
PanNIN.1
ATG
STOP
PanNIN.2
(b)
PanNIN.1
PanNIN.2
Normalized counts
500
450
400
350
300
250
200
150
100
50
0
a
a
b
c
c
c
c
c
Roots
Nodule primordium
Young nodules
Mature nodules
(c)
PanNIN.1 ATG
PanNIN.2 ATG
200 bp
PanNIN
(d)
PanNIN WT
Pannin.1 B1
Pannin.1 B3

Fig. S3: *Rhizobium tropici* CIAT899.pMP604 constitutively expresses the LCO biosynthesis gene *nodC*.

The pMP604 plasmid encodes an autoactive variant of *nodD*, a transcription factor that regulates LCO biosynthesis genes. Shown are the relative expression of *nodC* in wild-type *R. tropici* CIAT899 (white dots) and *R. tropici* strain CIAT899 transformed with pMP604 (black dots) in the absence or presence of increasing concentrations of naringenin (nM). Dots represent technical repeats.

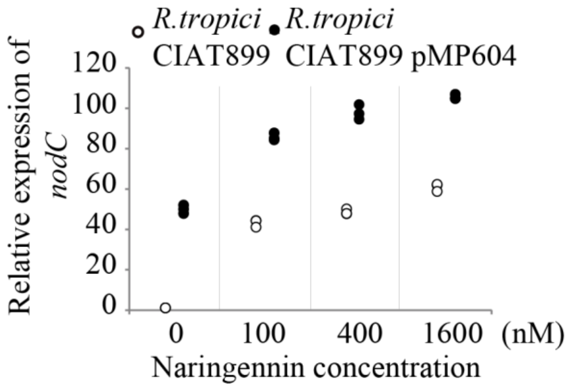

Fig. S4: Structure of the *P. andersonii NF-YA1* gene and genotype of CRISPR-Cas9 *Panf-ya1* mutants.

(**a**) Schematic representation of the *PanNF-YA1* gene model. Indicated are the locations of 3 sgRNAs used for mutagenesis (red triangles). (**b**) Genotype of the bi-allelic *Pannf-ya1-1* mutant line. (**c**) Schematic representation of the *PanNF-YA1* gene model. Indicated (red triangles) are the location of sgRNA used in an independent transformation to creating *Pannf-ya1* CRISPR-Cas9 mutants. (**d**) The genotype of the *Pannf-ya1* mutant lines m3 and m5. Shown is the sequence of the first coding exon.

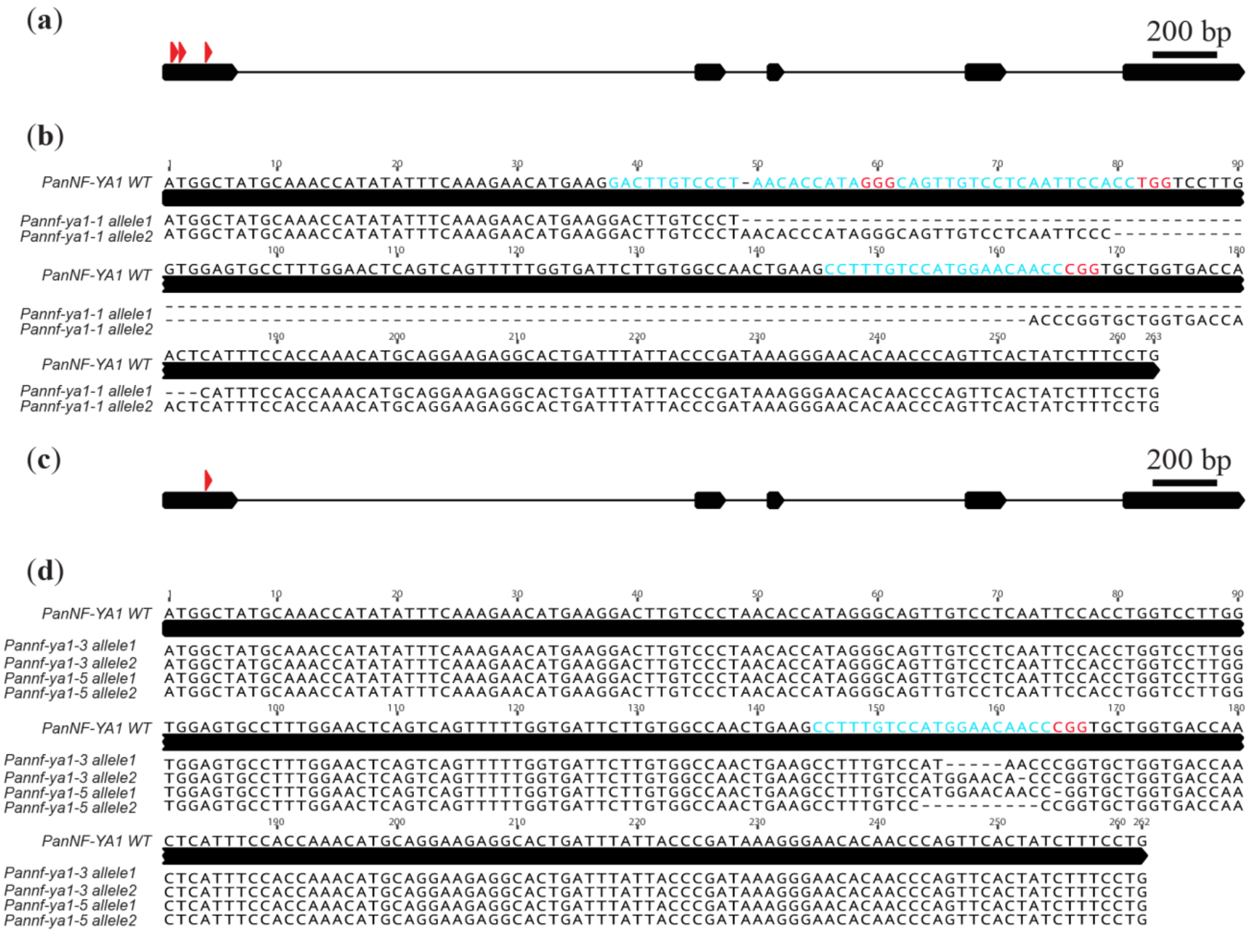

.

Fig. S5 Lateral root formation is affected in the *P. andersonii nf-ya1* mutant.

(a) Number of roots formed on plantlets incubated on rooting medium for 14 days is lower in *Pannf-ya1-1* mutants (n=67 for EV-control n=66 for *Pannf-ya1-1*). Statistical significance based on a student's T-test $p<0.05$. (**b-d**) Plantlets in a comparable developmental stage were selected to be used for the root formation assay, transferred to EKM-agar plates lined with cellophane and scored after 20 days. EV-control n=12, *Pannf-ya1-1* n=12. **(b)** Number of growing main roots on EKM medium is not different between EV-control and *Pannf-ya1-1*. Main roots are characterized as all roots directly attached to the shoot. Given the nature of rooted plantlets there are usually multiple roots. **(c)** Total summed main root length per shoot is not different between EV-control and *Pannf-ya1-1*. **(d)** Number of lateral roots formed on main roots is reduced in *Pannf-ya1-1* mutants. Statistical significance for (b,c,d) based on Mann Whitney U-test $p<0.05$. **(e)** Representative examples of EV-control plantlets grown 20 days on EKM-agar plates. **(f)** Representative examples of *Pannf-ya1-1* plantlets grown for 20 days on EKM-agar plates.

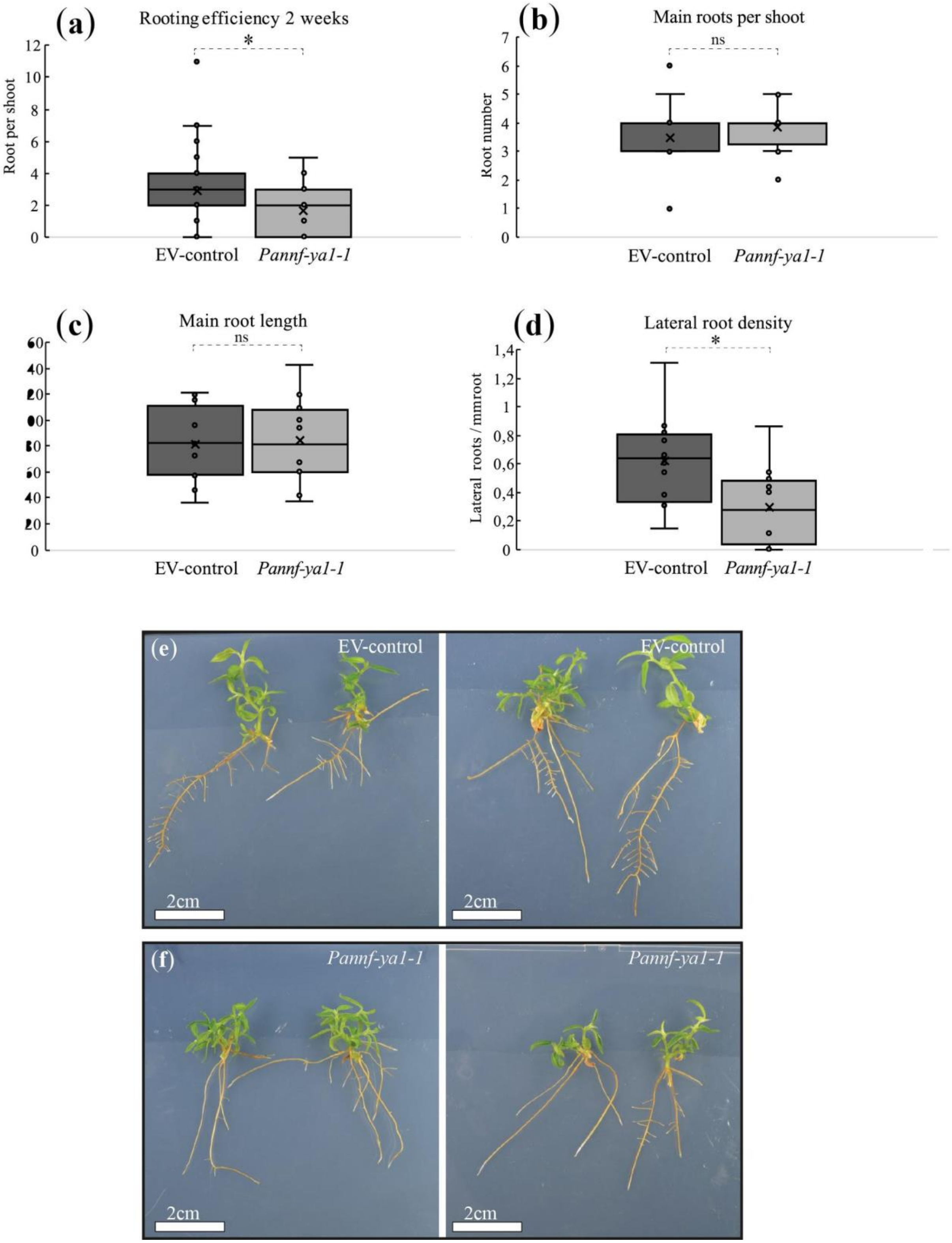

(a)
Rooting efficiency 2 weeks
*
Root per shoot
0
2
4
6
8
10
12
EV-control
Pannf-ya1-1
(b)
Main roots per shoot
ns
Root number
0
1
2
3
4
5
6
7
EV-control
Pannf-ya1-1
(c)
Main root length
ns
EV-control
Pannf-ya1-1
(d)
Lateral root density
*
Lateral roots / mmroot
0
0,2
0,4
0,6
0,8
1
1,2
1,4
EV-control
Pannf-ya1-1
(e)
EV-control
EV-control
2cm
2cm
(f)
Pannf-ya1-1
Pannf-ya1-1
2cm
2cm

Fig. S6: Phenotyping of *P. andersonii nf-ya1* knockout mutants.

(**a**) Average number of nodules formed on wild type (WT), transgenic control line CTR44, and the CRISPR-Cas9 knockout mutant *Pannf-ya1* (line *Pannf-ya1-1*), 5.5 weeks post-inoculation with *M. plurifarium* BOR2. Different letters indicate statistical significance (Student's t-test, $p < 0.05$). (**b**) Nitrogenase activity measured by an acetylene reduction assay (ARA) on nodules formed on transgenic control line CTR44 and *Pannf-ya1* (*Pannf-ya1-1*). Data represent means (n = 15 in panel a, n = 5 in panel b) ± SD. nd.: not detected. (**c**) Cytoarchitecture of nodules formed on *Pannf-ya1-3* and (d) *Pannf-ya1-5*, 4 weeks post inoculation with *M. plurifarium* BOR2. v: nodule vasculature; ac: apoplastic colonies of rhizobia.

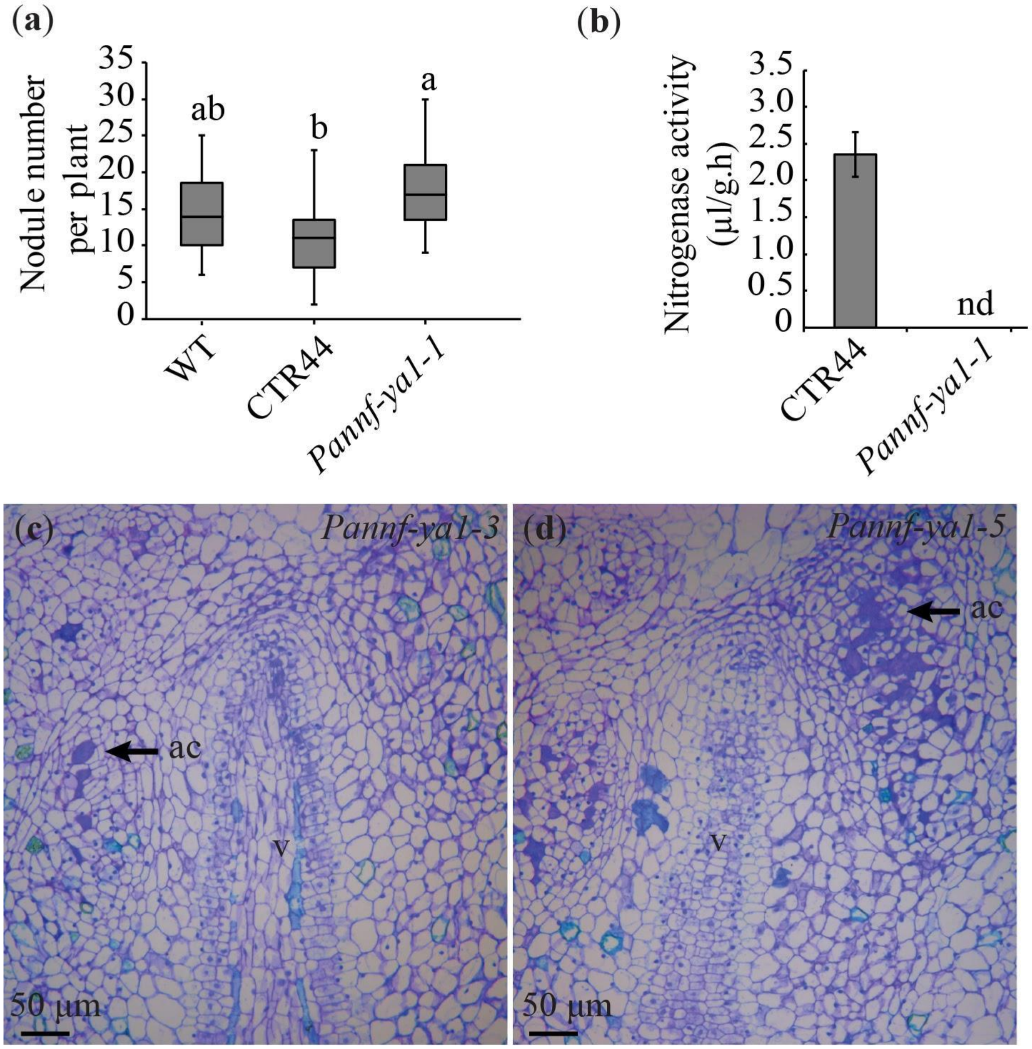

Fig. S7: Phylogenetic analysis of NF-YA in the nitrogen-fixing clade.

Bayesian phylogeny of NF-YA proteins reconstructed based on an alignment of protein sequences from the following species: *Parasponia andersonii* (Pan), *Trema orientalis* (Tor), *Arabidopsis thaliana* (At), *Medicago truncatula* (Mt), *Lotus japonicus* (Lj), *Glycine max* (Glyma), *Arachis duranensis* (Adu), *Phaseolus vulgaris* (Pv), *Casuarina glauca* (Cgl), *Datisca glomerata* (Dgl), *Morus notabilis* (Mno), *Prunus persica* (Ppe), and *Fragaria vesca* (Fve). *P. andersonii* NF-YA proteins are marked in red. Red pentagrams mark duplication events within the legume family. Orthogroups are indicated by coloured circles. Node labels indicate posterior probability, Node labels with a value above 0.9 are not shown.

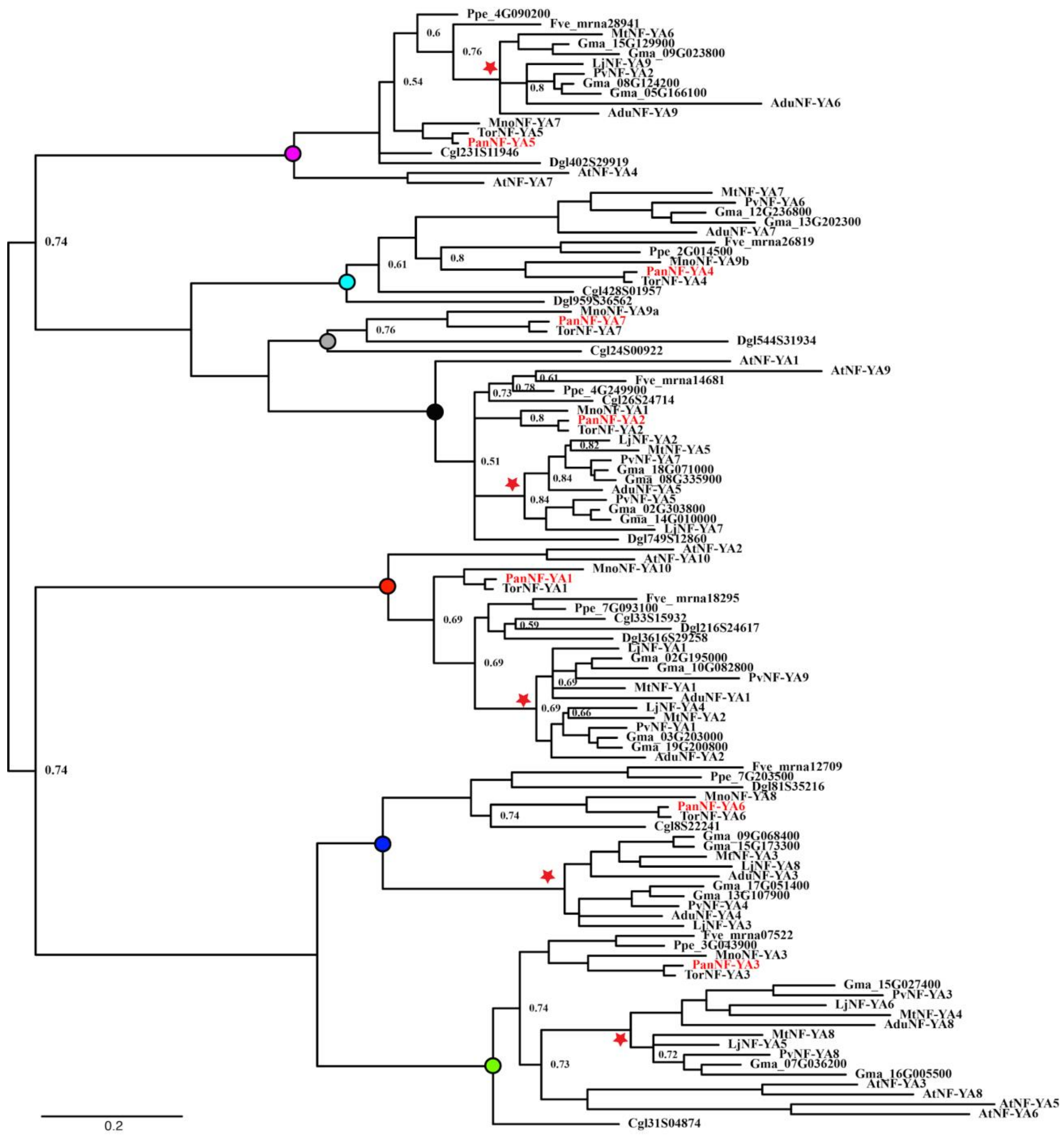

Ppe_4G090200
Fve_mrna28941
MtNF-YA6
Gma_15G129900
Gma_09G023800
LjNF-YA9
PvNF-YA2
Gma_08G124200
Gma_05G166100
AduNF-YA6
AduNF-YA9
MnoNF-YA7
TorNF-YA5
PanNF-YA5
Cgl231S11946
Dgl402S29919
AtNF-YA4
AtNF-YA7
MtNF-YA7
PvNF-YA6
Gma_12G236800
Gma_13G202300
AduNF-YA7
Fve_mrna26819
Ppe_2G014500
MnoNF-YA9b
PanNF-YA4
TorNF-YA4
Cgl428S01957
Dgl959S36562
MnoNF-YA9a
PanNF-YA7
TorNF-YA7
Dgl544S31934
Cgl24S00922
AtNF-YA1
AtNF-YA9
Fve_mrna14681
Ppe_4G249900
Cgl26S24714
MnoNF-YA1
PanNF-YA2
TorNF-YA2
LjNF-YA2
MtNF-YA5
PvNF-YA7
Gma_18G071000
Gma_08G335900
AduNF-YA5
PvNF-YA5
Gma_02G303800
Gma_14G010000
LjNF-YA7
Dgl749S12860
AtNF-YA2
AtNF-YA10
MnoNF-YA10
PanNF-YA1
TorNF-YA1
Fve_mrna18295
Ppe_7G093100
Cgl33S15932
Dgl216S24617
Dgl3616S29258
LjNF-YA1
Gma_02G195000
Gma_10G082800
PvNF-YA9
MtNF-YA1
AduNF-YA1
LjNF-YA4
MtNF-YA2
PvNF-YA1
Gma_03G203000
Gma_19G200800
AduNF-YA2
Fve_mrna12709
Ppe_7G203500
Dgl81S35216
MnoNF-YA8
PanNF-YA6
TorNF-YA6
Cgl8S22241
Gma_09G068400
Gma_15G173300
MtNF-YA3
LjNF-YA8
AduNF-YA3
Gma_17G051400
Gma_13G107900
PvNF-YA4
AduNF-YA4
LjNF-YA3
Fve_mrna07522
Ppe_3G043900
MnoNF-YA3
PanNF-YA3
TorNF-YA3
Gma_15G027400
PvNF-YA3
LjNF-YA6
MtNF-YA4
AduNF-YA8
MtNF-YA8
LjNF-YA5
PvNF-YA8
Gma_07G036200
Gma_16G005500
AtNF-YA3
AtNF-YA8
AtNF-YA5
AtNF-YA6
Cgl31S04874
0.6
0.76
0.8
0.54
0.74
0.61
0.8
0.76
0.61
0.78
0.73
0.8
0.51
0.82
0.84
0.84
0.69
0.59
0.69
0.69
0.69
0.66
0.74
0.74
0.74
0.73
0.72
0.2

Fig. S8: Expression of *PanNF-YA3* and *PanNF-YA6* in *P. andersonii* roots and nodules.

(**a**) Expression of *PanNF-YA3*$_{pro}$:GUS in uninoculated roots. (b-d) Expression of PanNF-YA3$_{pro}$:GUS following inoculation with *M. plurifarium* strain BOR2. (**e, f**) *PanNF-YA6*$_{pro}$:GUS in uninocualted roots (e) and following inoculation with *M. plurifarium* strain BOR2 (f). (**a**) *PanNF-YA3$_{pro}$*:GUS is expressed in the root meristem, (**b, c**) in discrete spots along the root (**d**) and in mature nodules. (**e**) *PanNF-YA6*$_{pro}$:GUS is expressed at the root meristem and root vasculature, and (**f**) the tip of the nodule. (**g**) Relative expression of *PanNF-YA3* and (**h**) *PanNF-YA6* in non-inoculated and *R. tropici* CIAT899.pMP604 inoculated (1 DPI) transgenic control (CTR44) and *Pannin* mutant (line B3) roots detected by qRT-PCR. Data were generated from the RNA samples used in Figure 2. RNA was isolated from root segments encompassing the elongation and part of the differentiation zone at 1 DPI with *R. tropici* CIAT899.pMP604. Data represent means of 2 independent experiments with a total of 5 biological replicates each ± SE. Data were normalized against the mock-treated CTR44 sample. np: nodule primordium; nodule: mature nodule.

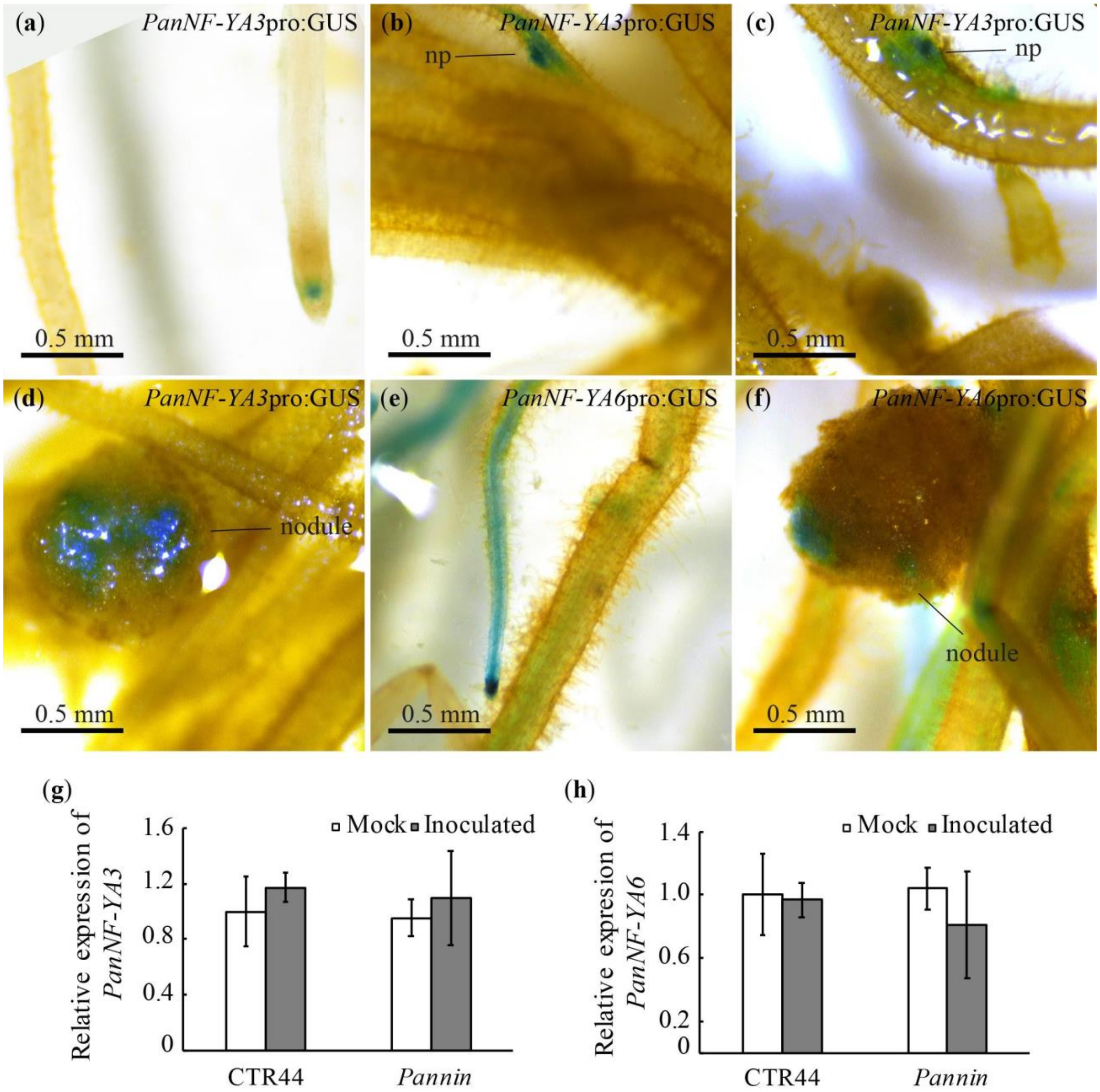

(a) PanNF-YA3pro:GUS
0.5 mm
(b) PanNF-YA3pro:GUS
np
0.5 mm
(c) PanNF-YA3pro:GUS
np
0.5 mm
(d) PanNF-YA3pro:GUS
nodule
0.5 mm
(e) PanNF-YA6pro:GUS
0.5 mm
(f) PanNF-YA6pro:GUS
nodule
0.5 mm
(g)
Relative expression of PanNF-YA3
Mock
Inoculated
1.6
1.2
0.8
0.4
0
CTR44
Pannin
(h)
Relative expression of PanNF-YA6
Mock
Inoculated
1.4
1.0
0.6
0.2
0
CTR44
Pannin

Fig. S9: Gene structure of *P. andersonii NF-YA3* and *NF-YA6*, genotype of CRISPR-Cas9 mutants, and nodulation phenotypes.

(**a**) Schematic representation of the *PanNF-YA3* gene model. Indicated are the positions of 3 sgRNAs used for mutagenesis (red triangles). (**b**) The genotype of the *Pannf-ya3* mutant lines 11 and 16. Shown is the sequence of the first coding exon. (**c**) Schematic representation of the *PanNF-YA6* gene model. Indicated are the positions of 3 sgRNAs used for mutagenesis (red triangles). (**d**) The genotype of 4 *Pannf-ya6* mutant lines. Shown is the sequence of the second coding exon. (**e**) Averaged number of nodules formed on wild type (WT), transgenic control line CTR44, and the *Pannf-ya3* (line 11) and *Pannf-ya6* (line 3) CRISPR-Cas9 knockout mutants at 5.5 weeks post-inoculation with *M. plurifarium* BOR2. (**f**) Nitrogenase activity measured by an acetylene reduction assay (ARA) on nodules formed on transgenic control line CTR44 and *Pannf-ya3* (line 11) and *Pannf-ya6* (line 3). Data represent means (n = 15 in E, n = 5 in F) ± SD. (**g, h**) Cytoarchitecture of *Pannf-ya3* (line 11) (**g**) and *Pannf-ya6* (line 3) (**h**) mutant nodules induced by *M. plurifarium* BOR2 (4 weeks post-inoculation). In both cases, nodules are indistinguishable from wild-type. sgRNAs are marked in blue, PAM sequences in red. m: nodule meristem; in: infection zone; fix: fixation zone; v indicates nodule vasculature. Scale bars are equal to 50 µm.

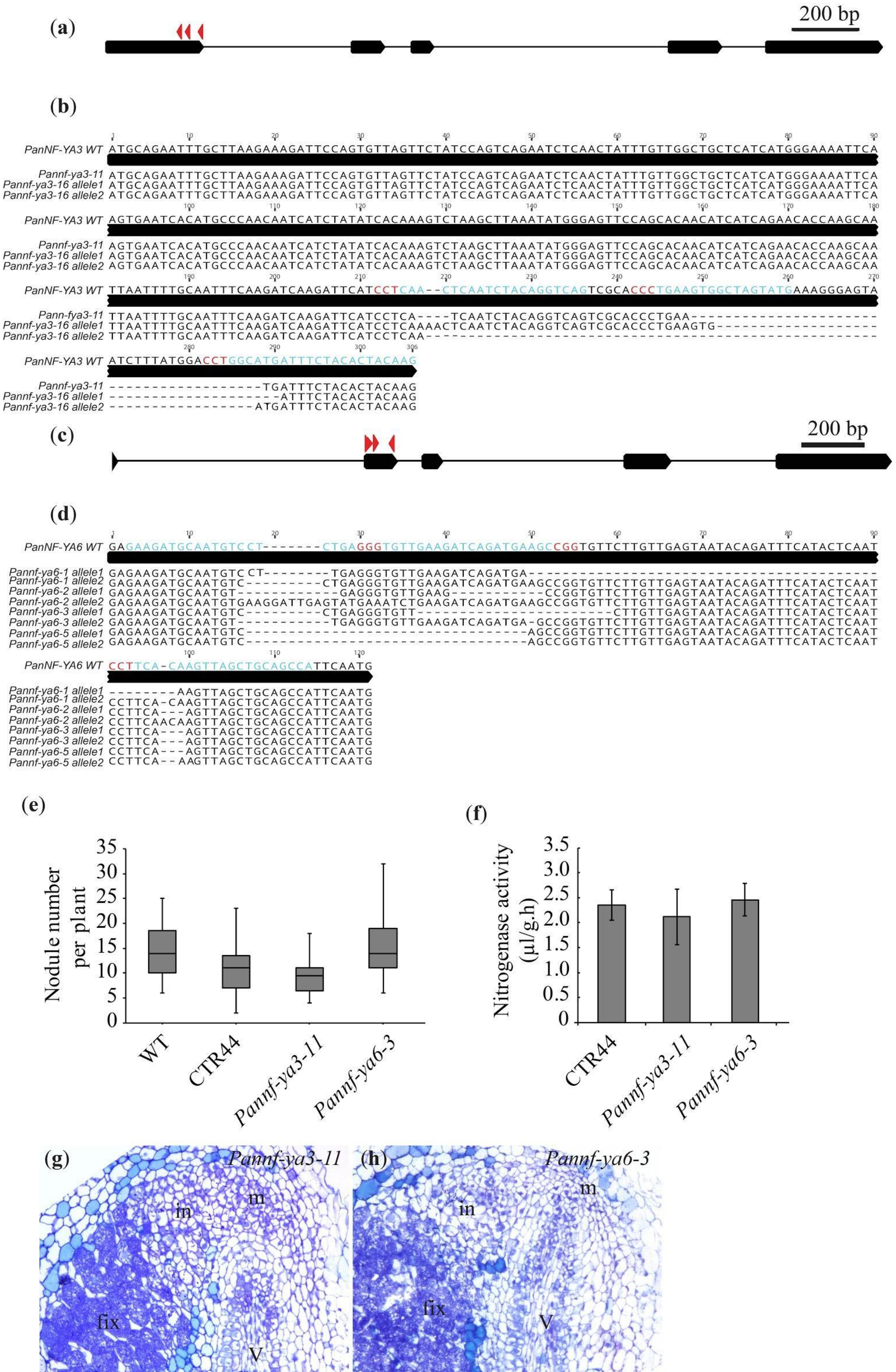

(a)
200 bp
(b)
PanNF-YA3 WT ATGCAGAATTTGCTTAAGAAAGATTCCAGTGTTAGTTCTATCCAGTCAGAATCTCAACTATTTGTTGGCTGCTCATCATGGGAAAATTCA
Pannf-ya3-11 ATGCAGAATTTGCTTAAGAAAGATTCCAGTGTTAGTTCTATCCAGTCAGAATCTCAACTATTTGTTGGCTGCTCATCATGGGAAAATTCA
Pannf-ya3-16 allele1 ATGCAGAATTTGCTTAAGAAAGATTCCAGTGTTAGTTCTATCCAGTCAGAATCTCAACTATTTGTTGGCTGCTCATCATGGGAAAATTCA
Pannf-ya3-16 allele2 ATGCAGAATTTGCTTAAGAAAGATTCCAGTGTTAGTTCTATCCAGTCAGAATCTCAACTATTTGTTGGCTGCTCATCATGGGAAAATTCA
PanNF-YA3 WT AGTGAATCACATGCCCAACAATCATCTATATCACAAAGTCTAAGCTTAAATATGGGAGTTCCAGCACAACATCATCAGAACACCAAGCAA
Pannf-ya3-11 AGTGAATCACATGCCCAACAATCATCTATATCACAAAGTCTAAGCTTAAATATGGGAGTTCCAGCACAACATCATCAGAACACCAAGCAA
Pannf-ya3-16 allele1 AGTGAATCACATGCCCAACAATCATCTATATCACAAAGTCTAAGCTTAAATATGGGAGTTCCAGCACAACATCATCAGAACACCAAGCAA
Pannf-ya3-16 allele2 AGTGAATCACATGCCCAACAATCATCTATATCACAAAGTCTAAGCTTAAATATGGGAGTTCCAGCACAACATCATCAGAACACCAAGCAA
PanNF-YA3 WT TTAATTTTGCAATTTCAAGATCAAGATTCATCCTCAA--CTCAATCTACAGGTCAGTCGCACCCTGAAGTGGCTAGTATGAAAGGGAGTA
Pann-fya3-11 TTAATTTTGCAATTTCAAGATCAAGATTCATCCTCA----TCAATCTACAGGTCAGTCGCACCCTGAA---------------------
Pannf-ya3-16 allele1 TTAATTTTGCAATTTCAAGATCAAGATTCATCCTCAAAACTCAATCTACAGGTCAGTCGCACCCTGAAGTG------------------
Pannf-ya3-16 allele2 TTAATTTTGCAATTTCAAGATCAAGATTCATCCTCAA---
PanNF-YA3 WT ATCTTTATGGACCTGGCATGATTTCTACACTACAAG
Pannf-ya3-11 -----------------TGATTTCTACACTACAAG
Pannf-ya3-16 allele1 --------------------ATTTCTACACTACAAG
Pannf-ya3-16 allele2 -----------------ATGATTTCTACACTACAAG
(c)
200 bp
(d)
PanNF-YA6 WT GAGAAGATGCAATGTCCT-------CTGAGGGTGTTGAAGATCAGATGAAGCCGGTGTTCTTGTTGAGTAATACAGATTTCATACTCAAT
Pannf-ya6-1 allele1 GAGAAGATGCAATGTCCT--------TGAGGGTGTTGAAGATCAGATGA---------------------------------------
Pannf-ya6-1 allele2 GAGAAGATGCAATGTC---------CTGAGGGTGTTGAAGATCAGATGAAGCCGGTGTTCTTGTTGAGTAATACAGATTTCATACTCAAT
Pannf-ya6-2 allele1 GAGAAGATGCAATGT-------------GAGGGTGTTGAAG-----------CCGGTGTTCTTGTTGAGTAATACAGATTTCATACTCAAT
Pannf-ya6-2 allele2 GAGAAGATGCAATGTGAAGGATTGAGTATGAAATCTGAAGATCAGATGAAGCCGGTGTTCTTGTTGAGTAATACAGATTTCATACTCAAT
Pannf-ya6-3 allele1 GAGAAGATGCAATGTC---------CTGAGGGTGTT------------------------CTTGTTGAGTAATACAGATTTCATACTCAAT
Pannf-ya6-3 allele2 GAGAAGATGCAATGT------------TGAGGGTGTTGAAGATCAGATGA-GCCGGTGTTCTTGTTGAGTAATACAGATTTCATACTCAAT
Pannf-ya6-5 allele1 GAGAAGATGCAATGTC------------------------------AGCCGGTGTTCTTGTTGAGTAATACAGATTTCATACTCAAT
Pannf-ya6-5 allele2 GAGAAGATGCAATGTC------------------------------AGCCGGTGTTCTTGTTGAGTAATACAGATTTCATACTCAAT
PanNF-YA6 WT CCTTCA-CAAGTTAGCTGCAGCCATTCAATG
Pannf-ya6-1 allele1 --------AAGTTAGCTGCAGCCATTCAATG
Pannf-ya6-1 allele2 CCTTCA-CAAGTTAGCTGCAGCCATTCAATG
Pannf-ya6-2 allele1 CCTTCA---AGTTAGCTGCAGCCATTCAATG
Pannf-ya6-2 allele2 CCTTCAACAAGTTAGCTGCAGCCATTCAATG
Pannf-ya6-3 allele1 CCTTCA---AGTTAGCTGCAGCCATTCAATG
Pannf-ya6-3 allele2 CCTTCA---AGTTAGCTGCAGCCATTCAATG
Pannf-ya6-5 allele1 CCTTCA---AGTTAGCTGCAGCCATTCAATG
Pannf-ya6-5 allele2 CCTTCA--AAGTTAGCTGCAGCCATTCAATG
(e)
Nodule number per plant
0
5
10
15
20
25
30
35
WT
CTR44
Pannf-ya3-11
Pannf-ya6-3
(f)
Nitrogenase activity (µl/g.h)
0
0.5
1.0
1.5
2.0
2.5
3.0
3.5
CTR44
Pannf-ya3-11
Pannf-ya6-3
(g)
Pannf-ya3-11
in
m
fix
V
(h)
Pannf-ya6-3
in
m
fix
V

Fig. S10: Genotypes of *Pannf-ya1, Pannf-ya3* and *Pannf-ya6* CRISPR-Cas9 double and triple mutants.

(**a**) Schematic representation and (**b**) genotype of *PanNF-YA1* for double knockout mutant *Pannf-ya1;Pannf-ya3* (line m1), *Pannf-ya1;Pannf-ya6* (line m6) and *Pannf-ya1;Pannf-ya3;Pannf-ya6* triple mutants (line m7, m8 and m11). (**c**) Schematic representation and (**d**) genotype of *PanNF-YA3* for double and triple knockout mutants. Notice that a different number of sgRNAs targeting *PanNF-YA3* were used to create double mutants (2 sgRNAs; DM) and triple mutants (1 sgRNA; TM). (**e**) Schematic representation and (**f**) genotype of *PanNF-YA6* for double and triple knockout mutants. Red triangles indicate the positions of sgRNAs used for mutagenesis. Shown in b and d are the sequences of the first coding exons, shown in f is the second coding exon. sgRNAs target sites are marked in blue, PAM sequences in red.

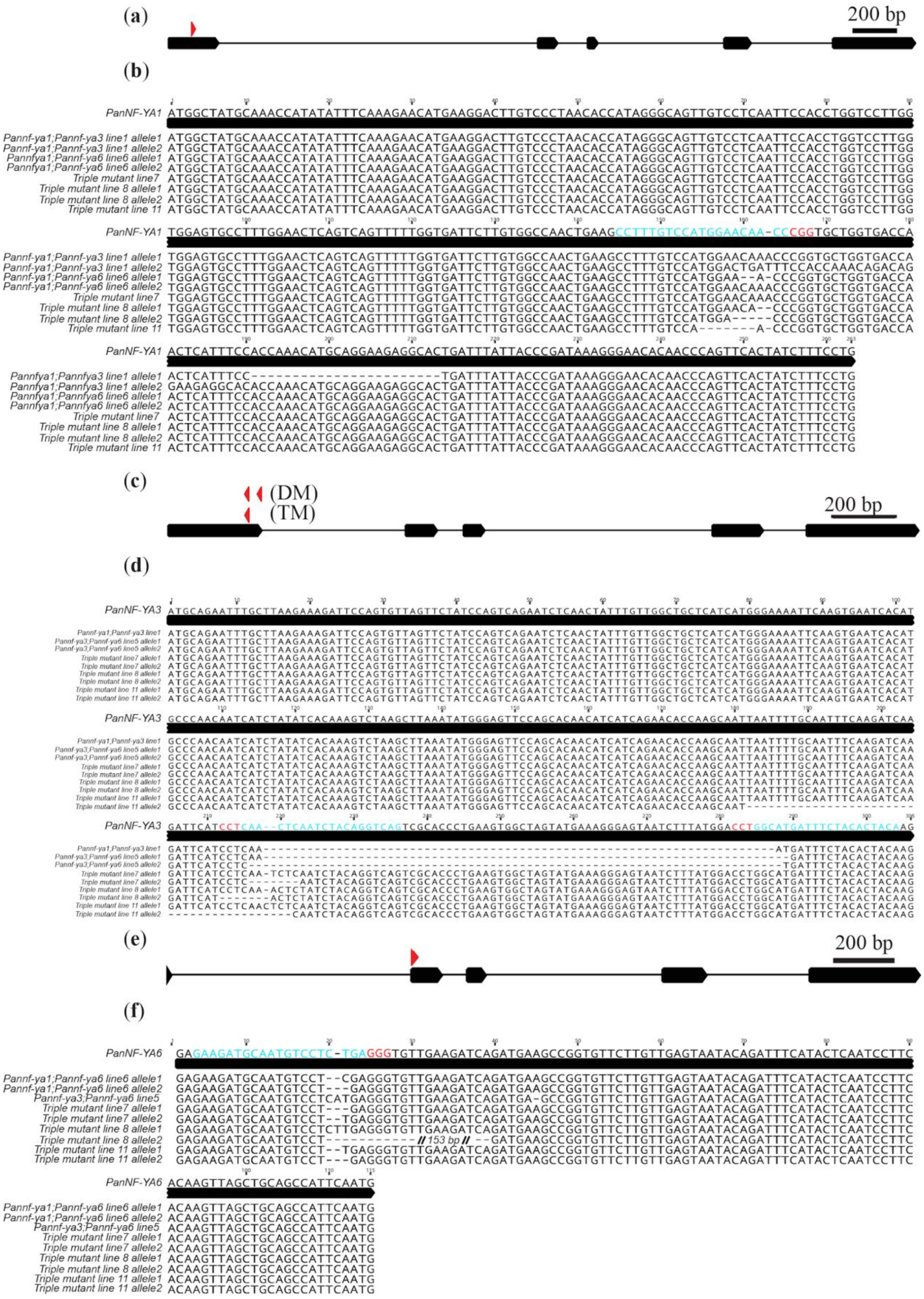
(a)
200 bp
(b)
PanNF-YA1
(c)
(DM)
(TM)
200 bp
(d)
PanNF-YA3
(e)
200 bp
(f)
PanNF-YA6
153 bp

Fig. S11: Nodulation efficiency and nodule size of *Parasponia andersonii nf-ya1* single, double and triple knockout mutants.

(**a**) Average nodule number per 100 mg root fresh weight, formed on transgenic control line CTR44, and CRISPR-Cas9 knockout mutants *Pannf-ya1* (line *Pannf-ya1-1*), *Pannnf-ya1;Pannf-ya3* (line 1), *Pannf-ya1;Pannf-ya6* (line 6), *Pannf-ya3;Pannf-ya6* (line 5) and the *Pannf-ya1;Pannf-ya3;Pannf-ya6* triple mutant (line 11), 5.5 weeks post-inoculation with *M. plurifarium* BOR2. (**b**) Nodule size presents averaged nodule size measured by nodule area (2D). In the case of the *Pannf-ya1;Pannf-ya3;Pannf-ya6* triple mutant, only nodule structures as shown in Figure 6B were included in this analysis. Data represent means (n = 4-5 plants) ± SE. Different letters indicate statistical significance (Student's t-test, $p < 0.05$).

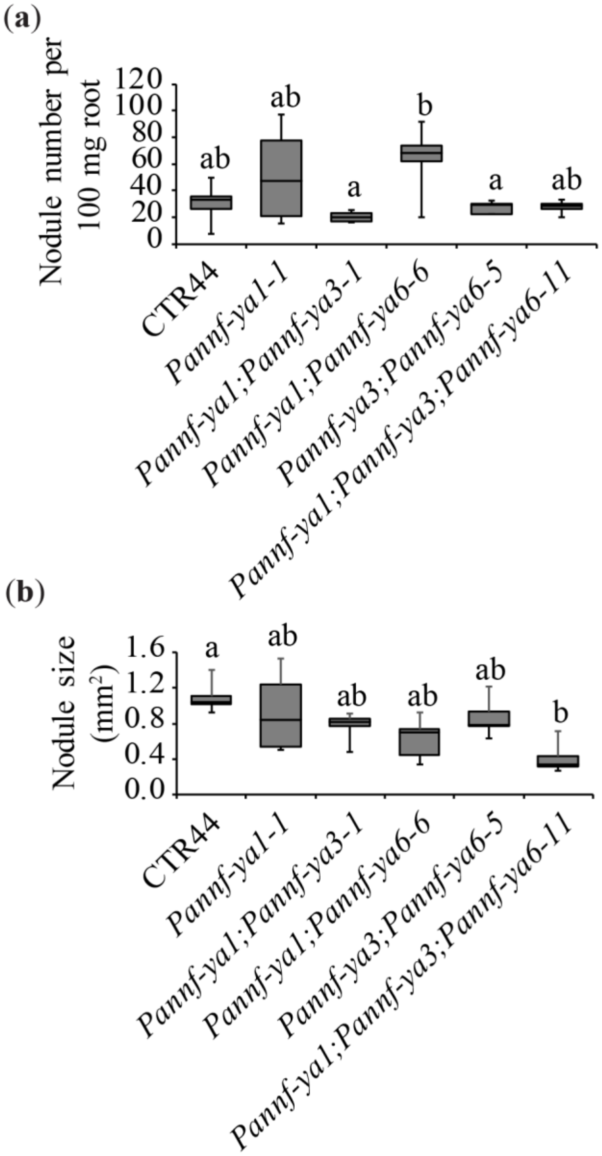
(a)
Nodule number per 100 mg root
120
100
80
60
40
20
0
ab
ab
a
b
a
ab
CTR44
Pannf-ya1-1
Pannf-ya1;Pannf-ya3-1
Pannf-ya1;Pannf-ya6-6
Pannf-ya3;Pannf-ya6-5
Pannf-ya1;Pannf-ya3;Pannf-ya6-11
(b)
Nodule size (mm2)
1.6
1.2
0.8
0.4
0.0
a
ab
ab
ab
ab
b
CTR44
Pannf-ya1-1
Pannf-ya1;Pannf-ya3-1
Pannf-ya1;Pannf-ya6-6
Pannf-ya3;Pannf-ya6-5
Pannf-ya1;Pannf-ya3;Pannf-ya6-11

Fig. S12: Nodule cytoarchitecture of *Parasponia andersonii nf-ya* double knockout mutants.

(**a, d**) Sections of nodules formed at 5.5 weeks post-inoculation on *Pannf-ya1;Pannf-ya3*, (**b, e**) *Pannf-ya1;Pannf-ya6* and (**c, f**) *Pannf-ya3;Pannf-ya6* mutant plants. (**d, e)** A zoom-in of nodule shown in a and b to visualize the absence of rhizobium intracellular infection threads. (**f**) A zoom-in of the nodule shown in c to visualize normal rhizobium intracellular infection. in: infection zone; fix: fixation zone; v: nodule vasculature; it: intracellular infection thread; ic: infected cells; nc: non-infected cells; ac: apoplastic colonies of rhizobia.

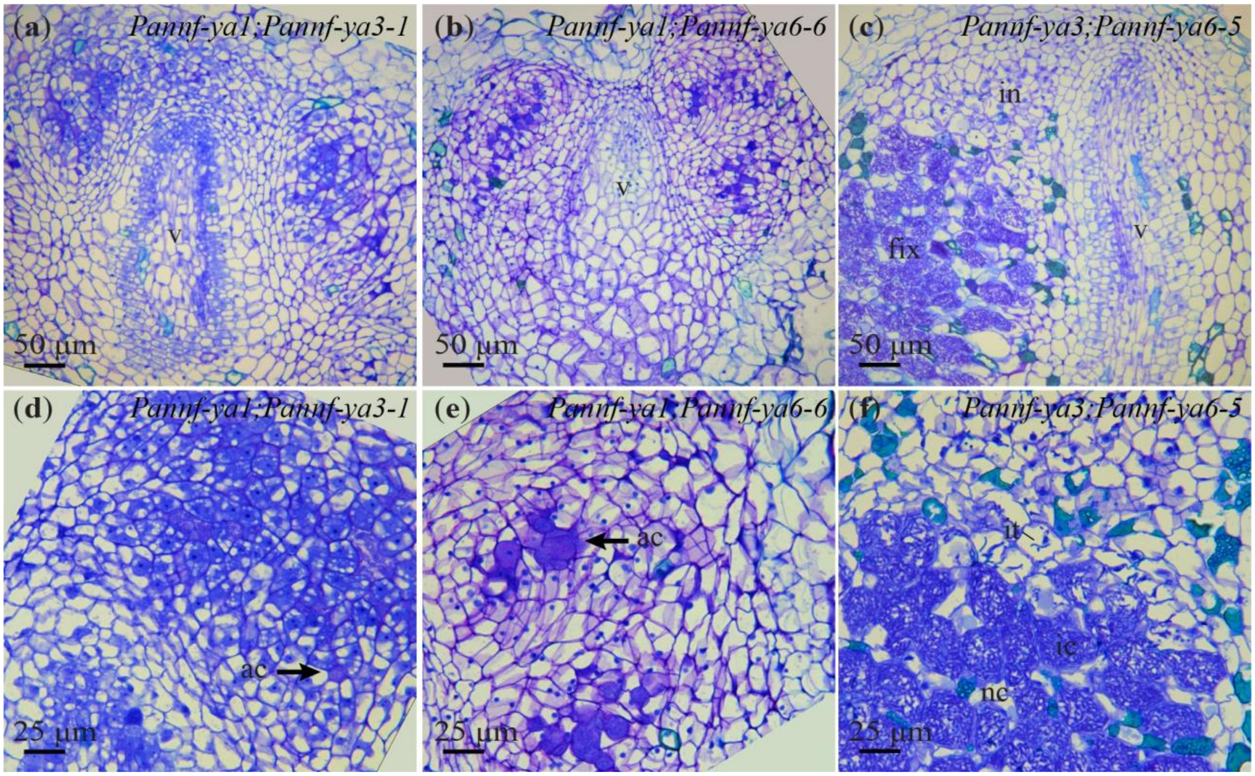

Fig. S13: Casparian strips in the vascular endodermis next to the nodule meristem in *Pannf-ya1;Pannf-ya3;Pannf-ya6* mutant.

(**a, b**) Visualization of Casperian strips in nodule sections of transgenic control (CRT44) (**a**) and *Pannf-ya1;Pannf-ya3;Pannf-ya6* triple mutant plants (**b**). Note that casparian strips (white arrows) are not present in the nodule vascular tip in control (**a**), but present in the vascular tip of the mutant nodule (**b**). Casparian strips are detected as auto-fluorescence under UV light. v: nodule vasculature. n = 5 nodules per lines.

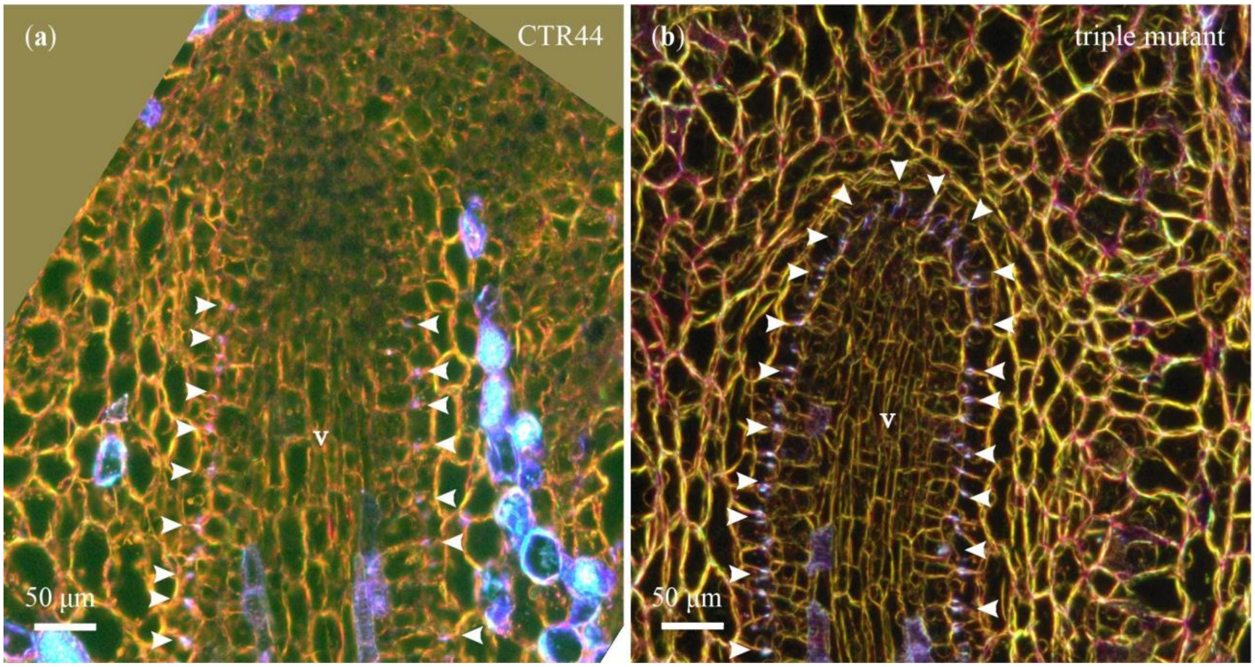

Fig. S14 ***Parasponia andersonii nf-ya1, nf-ya3, nf-ya6*** **and** ***Pannf-ya1;Pannf-ya3;Pannf-ya6*** **mutants can form arbuscular mycorrhiza.**

Mycorrhization efficiency of *P. andersonii* wild type (WT), transgenic control line CTR44, and CRISPR-Cas9 knockout mutants *Pannf-ya1* (line 1), *Pannf-ya3* (line 11), *Pannf-ya6* (line 3) and *Pannf-ya1;Pannf-ya3;Pannf-ya6* triple mutant (line 11), 6 weeks post-inoculation with *Rhizophagus irregularis* DOAM197198 (n > 5). Error bars denote standard errors.

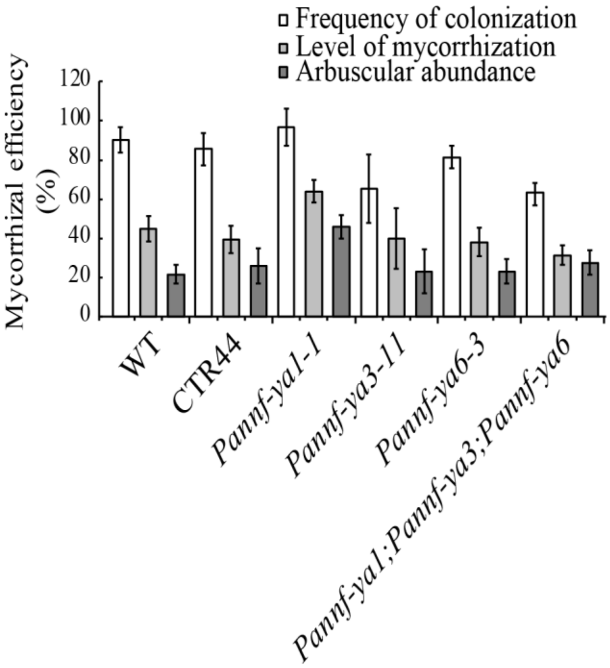

New Phytologist

Fig. S15: Putative NIN Binding sites in the *PanNF-YA1* promoter region.

Shown is the annotated *PanNF-YA1* gene, including the putative promoter region (2,823 bp) and 5'UTR (977 bp containing an intron of 643 bp) that was used for *PanNF-YA1*$_{pro}$:GUS reporter studies. A single putative NIN binding site with sequence TTAACTTTTAGATGCTAGGTAGGGTTAATT 1,785 bp upstream of the transcriptional start site- is predicted based as the distinct consensus sequences as defined by Soyano et al. (2013) and Soyano et al. (2015).

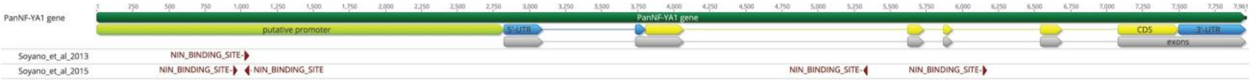

Table S1: Sequences of sgRNAs used for creating single, double and triple knockout mutants.

sgRNA name	Single mutant	Double mutant	Triple mutant	Location	Genomic target sequence
PanNIN.1 sgRNA1	Y	-	-	exon 1	GATGTTTCGATGAGTGACCG
PanNIN.1 sgRNA2	Y	-	-	exon 1	ATGAGTTGTGATCAAGTGAG
PanNIN.1 sgRNA3	Y	-	-	exon 1	**A**TATGGGTGCCCATTAGAAG
PanNF-YA1 sgRNA1	Y	-	-	exon 1	CAGTTGTCCTCAATTCCACC
PanNF-YA1 sgRNA2	Y	Y	Y	exon 1	CCTTTGTCCATGGAACAACC
PanNF-YA1 sgRNA3	Y	-	-	exon 1	GACTTGTCCCTAACACCATA
PanNF-YA3 sgRNA1	Y	-	-	exon 1	TGTAGTGTAGAAATCATGCC
PanNF-YA3 sgRNA2	Y	Y	Y	exon 1	CTGACCTGTAGATTGAGTTG
PanNF-YA3 sgRNA3	Y	Y	-	exon 1	CTTTCATACTAGCCACTTCA
PanNF-YA6 sgRNA1	Y	-	-	exon 2	TGTTGAAGATCAGATGAAGC
PanNF-YA6 sgRNA2	Y	Y	Y	exon 2	GAAGATGCAATGTCCTCTGA
PanNF-YA6 sgRNA3	Y	-	-	exon 2	TGGCTGCAGCTAACTTGTGA

Table S2: Primers used in this work.

Primer name	Primer sequence	Purpose
sgRNA universal Reverse primer	TGTGGTCTCAAGCGTAATGCCAACTTTGTACGTTTTAGAGCTAGAAATAGCAAG	Cloning of sgRNAs
PanNIN.1 sgRNA1 F	TGTGGTCTCAATTGATGTTTCGATGAGTGACCGGTTTTAGAGCTAGAAATAGCAAG	
PanNIN.1 sgRNA2 F	TGTGGTCTCAATTGATGAGTTGTGATCAAGTGAGGTTTTAGAGCTAGAAATAGCAAG	
PanNIN.1 sgRNA3 F	TGTGGTCTCAATTGATATGGGTGCCCATTAGAAGGTTTTAGAGCTAGAAATAGCAAG	
PanNF-YA1 sgRNA1 F	TGTGGTCTCAATTGCAGTTGTCCTCAATTCCACCGTTTTAGAGCTAGAAATAGCAAG	
PanNF-YA1 sgRNA2 F	TGTGGTCTCAATTGCCTTTGTCCATGGAACAACCGTTTTAGAGCTAGAAATAGCAAG	
PanNF-YA1 sgRNA3 F	TGTGGTCTCAATTGACTTGTCCCTAACACCATAGTTTTAGAGCTAGAAATAGCAAG	
PanNF-YA3 sgRNA1 F	TGTGGTCTCAATTGTGTAGTGTAGAAATCATGCCGTTTTAGAGCTAGAAATAGCAAG	
PanNF-YA3 sgRNA2 F	TGTGGTCTCAATTGCTGACCTGTAGATTGAGTTGGTTTTAGAGCTAGAAATAGCAAG	
PanNF-YA3 sgRNA3 F	TGTGGTCTCAATTGCTTTCATACTAGCCACTTCAGTTTTAGAGCTAGAAATAGCAAG	
PanNF-YA6 sgRNA1 F	TGTGGTCTCAATTGTGTTGAAGATCAGATGAAGCGTTTTAGAGCTAGAAATAGCAAG	
PanNF-YA6 sgRNA2 F	TGTGGTCTCAATTGAAGATGCAATGTCCTCTGAGTTTTAGAGCTAGAAATAGCAAG	
PanNF-YA6 sgRNA3 F	TGTGGTCTCAATTGTGGCTGCAGCTAACTTGTGAGTTTTAGAGCTAGAAATAGCAAG	
PanNIN.1 geno F	CTCAACTTCACGCAACCTGC	Genotyping of CRISPR mutant lines
PanNIN.1 geno R	TCCCACGCTCAAAAATGGGA	
PanNF-YA1 geno F	TCCCCCTATTTTGGTCTTAGTCT	
PanNF-YA1 geno R	TGCAAACAACAGAGTTATAGGCC	
PanNF-YA3 geno F	CCAGCACAACATCATCAGAACA	
PanNF-YA3 geno R	TTGTTAAGCAACGTAGGGAACT	
PanNF-YA6 geno F	ATCTGGGTGGACAGGCAATG	
PanNF-YA6 geno R	CTTACAAGAGCCCGTGGTCC	
PanEF1a qF	AGACAAGGTTAAGCGTGCAG	qRT-PCR
PanEF1a qR	TGCAACTGGGCAACAAACTC	
PanNIN qF	TGGGAATGGGACTTGTTTGG	
PanNIN qR	GGGAGGGCTGAAGTTTTGAA	

PanNF-YA1 qF	CAGTCATCCCTGCCAGAATATC	
PanNF-YA1 qR	TGCAGTCAAGTTCAGCGG	
PanNF-YA3 qF	TCCCGCTATGATTCACCATTCC	
PanNF-YA3 qR	ATTGCACGGTACTGCTTTGC	
PanNF-YA6 qF	CCATTCAATGGCTGGTGTGC	
PanNF-YA6 qR	TCCAAAGGCAATGGAACTCG	

Table S3: Putative promoter sequences used for promoter-reporter GUS assays.

In red: nucleotide mutations to remove BsaI and/or BpiI restriction sites, which is essential for GoldenGate cloning; in blue: putative NIN binding site; underlined: 5'UTR; yellow highlighting: exon sequences in 5'UTR, in small letters: intron sequences; in bold: translational start site.

>*PanNF-YA1*$_{pro}$ (PanWU01x14_284830)

ATCGAAGCCTCCAAAAGGGGGGCAGAGTTATTAAATGATGAAGAGAATTTTTAGGTCACCTTAA
GATGTGGAAATTAAAGGTTCGATGTAGCACAATGTAAGCATACATTATGTTAGTCATGATGTTA
GTCACTAATTACAAATAGTTGTACTTGTATTGGTCATATCAGTCGATCTATGCTATTACATTGA
ACATTTGACATAAAATCCCAATATAATCCATATATTTAATGCTAAATTGACAAAATTATTACCA
ATATATATTCCTACTCTAATAGGTATTTTTTTTTACCAATTTATGTGTATTCTTTTTAACCATT
GGATCATTATCTAAATATTATTATAATTTGGATAAGTGGAAGAAAAACAAAAATAAATTAGGTA
AAACAATACGTATAAATAGATAAAAAAAATGAGAAATTTAGGTGGATGACTAATATTTTATATT
TGATTAAGTAGATATCTATTTTTTAAATTTTATAGCTAAATATCTAGGTATATATATTTTTATA
CTTAGTAGATACATTAATTTACCAATTTTAGCTACTGTACTGAGATGTACTGGATATAAAATAT
TTTATATTTAGTATATAAAATTTTACACTTGATATATAAGTATATATACCGGTTTATTATAATA
TTTGATAAATAGATATTTACTTAAATTAGGCTTAAAATTTGATCCTGTTTCTATAAGCCCCAAA
AAAATACCTATTGAATCAGCGTCCATATATTTATATATATACAAATGACCAAAAAAAAAAAAAA
CAATTAGAACATGAAACTACTAATGTTTATTGGCCAGAACCAAGAAAGTTGCAACACTTTTGTA
TAGGAATAATCTGGTTACAAGGTC**G**TCAGATTCCTATTTACATACTTTAATAAATGCATTTCCA
CCAAAAAAAATAAGAAAACAAAAAGCTCATTCCAATCTCAGCTAGTCTCTCTGCCCTTCTCAAA
TGACTAAGGGTCAGGACACTTTCACGAAATATAAGCTTTAGTTTTGGGGTTTACTTCATATAAA
GTTAACTTTTAGATGCTAGGTAGGGTTAATTACAAAGATTAAAAAAATGACAATTATAAGTGGA
AATGACCGTGACAGTGATAAATATTCAACACCATTAATTATATATACAATTGCTAAGCTAATCA
GAACTTTTCACGCATATAAAATACAATATTCTCATCCCAAATTAAGATTTTTATATTTGAAATG
TGACATAATAGTAACATGTTTTTTTTGGTCGTATACATTTATATATATGGTTCCTAGTAGGGAT
TTTTTTTAACTTATCTATAACTATTTTTATTTTTAGTCATTAGATGATGATCTAACGATTATTA
TAATTTGAGTAGTTAAAAAAAATAATATAAATGAATACTCATTATTCAATTCTTTTCTCTTTCT
ATCTTTAAAACATGTGTGATGATTTTTAATTAGGAAAAATAATACTTATGTATAGGTAAAAAAA
AATACCTACTAAATATATATATATATATATTTGAACACTAATCCAGTAGGTTTTTTTTTTTTACC
TATTTATAGGTATTCTTTTGTCCAATAAAAATTCATCACACGTATTTTTTATGATAAAAAAAAA
AGAGAGAAAAAATTATTAAATAATAAGTTTTTATTTTTTTATTTTTTTTTTATTTTTGATTGATA
ACCTATTTATATATTTTCCCACTACGGCCTGCTAATTAGCAACAGCCATAATCTTGACTGTCAT
TAAGTATCTTTAGCCATTAGAATTATGGTCATATATATCTTCAAGCTGTTAATTTGCACCACCG
AATGAATATTATAGTAATATCATTTAAGCTGAAAATATTACTATACTATGTTGGAGCATTTGCC
AACTCAATTTTTTCCCACTACTTCACTTGAGTGAAAGGGTAAATTTTTAAGTGATACTGATCCG
GCCTCTATGAATATGTTAGCTAATTATACTAAGGTAATAATAGGAACTAAAGATTGATATCAAT
GGTTATAAATATTGTTTGGAATGTTTATGTACACATCACTGAAAAAATTTTCTGAAATTGTTTT
TCTAACACGTTTGTGTTCATGAGGAATAAAAAAAAATTAACATTAACAAAAAAAAGGACACTAA
GCTGTTTTAATTTTTAAAAACCGGAAAAAATAAAATTATGGTATATATATACAACCTAATCCCA
CATGCCCTGACACTCCTTTTTATTTATTTATTTTTTCAAGAACAGAAAAAAGAATTTTGAGTAG
TAGGAGAAAAGTCTATTTTCTGACAGTTATATAATGTTTAAAATTAAACAATACTCAAGTATAT
AAAAATAACAAAGCAGTTTAAGGATTATAAACTATTTATATAAATAGTATTAAAAAATGGAAAT

GAAAACTATGACATCCCATCGGACCATGGACATGCCCTGGAATTAGAGAACCAAAGGCAAAAAC
CATGCACTCCACAACTCCTAAGCAAAATTTTTAACCTTATCCGCATTATTTTCTTAAACTTATC
TGGAACCCACTTGTATTATATACATATAATAAACAATACGAATTTTACATTACATACATATATA
TATTATATATATATATATATGGCAGGCTTTTGTATATTATATATAGTAATGAATAATCCAGTTT
TTTTTTTCCCACTGTGACATTCCAACACCCCAAGTTGTGAAGCTTAAGAAAATATTAAAGAGAA
AGGGGTACCTCAACTTTAGCTTAATTTGATAAAAATAAACATAAATAAGAATTGATCAAAATAT
GTTGGAGAAAAACGGTGAACCCGAGAGAGAAAGGGAGCACTAAATTCTTTTGTTTTATTTATTT
TTTTAAAAAAATATATATATACATATTATATAATTTGATCTGTAACACTATACATATTGGCACA
TACAATACAAAGAGGGGTACTTGCATGAAAGGCAAAGCTTATTGGCCATTGGCGTCTAGTTCCA
TTAGAATTGTACGCATTTATCATTAGGGGGTCGTCTCTCTCTCTCTCTCTCCTTCCCCATCTCT
CTCTCTCTCTCTCTGTTCTTGAGCGTTGGACACGAAGCAAACTGTTGCTGGGTACTGCTATTCA
GCTAGTTTTCTCTCATATgtgagctctcaaataccaaactttttttctattcttagccttgttgc
tccaacatagttggagggtcttgtcaaagtttcttttttttcttttaaagcttgaattttttttct
cccttctcatttttgatttctgggttttttttttgttctgagttgatgcttttgttcttttttt
tttttcccttcttttttaaatgtatctgtcagttaaagaagatgtaatttcagtgatcaaacag
acagaacagaattgtttgtaaccaatatttgatcaaaataaattttgagagctcattcacaatt
ttcatttttttttttcttcaatgaaacaccgtggttttcatcttttttattgtttcatttatatcg
acaatttattttttttcataatctcgaatcaaatcaattttgatcacttattatccttctgtat
gagtctgctaatcagaccagaattttttttttttgggccaaattttagttcaatattgaaactc
aatgaaatttatttcccccctattttggtcttagtctttgtcaactatagtatttttgtcatatt
tttcaatctcgtgtttacatagaatgttgttagatatagtcttatagactcataattaaggttg
gtgggttttcatatttgtagGCGTTAGATTCAACAATTGGTTTGAAGCTAGCTAGGTACTGTGG
TTTTGATCATTTTGCAAGTTAGCT**ATG**

>*PanNF-YA3*$_{pro}$ (PanWU01x14_246880)
CATAACGAAGAAACTCTTGGAGTAATTAGAAGGTTGGTGGTGGATGATGGGTTGCTTTCAAATT
TTGTATTTTTATTTTTTAAATGCGACTTTCAAGGTCATTTTATATTGACAGCAGATAAATGACA
TTGATTATATAAAGTAAGCTAGCTAATTCAGGGCTCTTTTGTAATAAATGCTGTGTAGAGGGTC
ATTAATTTAAAAGCTGCCATATTATCTTCCTATTAATTGCATTAATAAGTTTTATTTAATTAAT
TGAAATCCATGACAAAGATGGATATCTAGCTACCTTCACTCTCTTGGAGCGCGTGTGGAAAGTC
GTAAGAGAATATTAGAATTAGATTTAGAAGTACATGAGAATTCTTGGAAGCTCCTCATCCTCAT
TCCAGAGTAATGGGGACATGATTCAACTTCTTACATGTTATCCAAAAGCATCACACAAAAAAAT
TCAGTTGGTAAATTTAATTTTTTAAATTTGATCATGAGTTTATTCTAAAAACAAATTAATGCAA
AAAAAAAAAATTCTTATGTCTAGATTTTCTGTAATTTAAATATTAAATTTTGGAATCTGTCAAA
AAAATATATTTGTATTTTAAAATTTTAAACATTTGTATCCTCTATTCTTATTTTGTTAGTTCAA
CAATATTGTTTTTTTGTAAATATTTTAATTTTTTAAAAATAGAAATAACATAGTTTGGATTTGT
AATTTTTTGAGAAATAAATTTATCAAAATATGGGCATAGGATACAAATTTTTTTTACGAATTTC
AAAATGTAGACATTTTTTTTGGGTAGAAATTTAGGATTTAAACCGCATAAGGACTACTAGATTT
CTTGGAATTTTTTGCAGTTAACCCAAAAAAAATGACCATGAGTTGATTACTAATTTAGACCACT
AATATTTCTTTTTCTTAAGATAATAGTTGCATTTAGGGTAAAATCTTGTATTTGAAATTGAATG
CATTTTTTTTGTTGTTAAGAAATAGACTAAGATAATATGTGGTGTTGAATACCCTATAATTATA
TGGTCTTTAATATTATTCTTTTGTTAATTGCATTCATTTCGCAAGCTGTAAAATGCTTTCTACC
TATATCAAAAAAATTAATAAGAGATAATTTATAACCTTTAATTTATTTTCATGTAAGCGATTAA
ATTTCTCTTAGCCTTATCATTTCTGAATTATTCAAATGAATCTAATTAAACTATAGGTCTTATT
ATTAGAAAAAGTTTATATAATAGAAAAAACATAAAGGGTTTTTACTAAACGTAAAGTTAAAATT
AATAACTTCAAAAAAAAAAAAATTTACCTTAAAACAAATTAAAAAAGAAAAAGAAAAAAAAGAAA
ACAGGGGAGATAGTTGATTGATTTCAATGGGCCCTTGTAGCGCGTGGGTAGAACACTGGCCGTA

ATATCAAGTGTGGTGACAACTTAGTATTTATTGTACCAGAAACCAGAAAGCGTGGGAAATTCAT
ATTGTCGATTTACAGCAAAATTTATAATCTCTCGTACTTAAAAATATATATAAAAAATAAATAA
TAAAGAATAGAATTTTATAATCATTTGGTGCTACACGACAAACACAAACTACAATTTAATATTA
TGTTATTAGAATAATACGTATTATAATATACATATGTATATGTATACATCTAGTAAATGGGAAA
AATGTAAGATTGGATTGATTGATATAAATTTTAATTAAGAATAGCAATAATAATAATAGAAGAA
GAAGAATAAAAAAAAATAAAAACTCGGAGAAGGTGGGAAGGTGGTGGAGATGCTGGTGAAGTCC
CTAGTGAGTTTAATGCAATAAGAATTGTTTAAGGAGAGATTCAGTCTCAGAATTGACACCAAAA
GCCAAGTGTGCGAGAGAGAGAGAGAGAGAGAGAGAGAGAGCTTTGTGGGGTTAAACAATAAGAGAA
GAAGAGGAAGAGGGACATGCACAGTTCCAAGTCTACAACGATTCCCATTTCTTGTTTTTCCACAC
TTTACTAAATCCAAAAAAGGTCCTTCCTTTACCCAAAGgtttatctttttctctctactttctt
gccttttcttcaactgggatttcttcttcttcttcttgttctctctcctcctttttttttttgtg
tccttatttttttgtttttaaactgcccattttatgtctgctactttttttgttccctcgcg
gatatcgttgtgtggcagtgaatgttacatgagattgcatgtggagacttaagatggtaaagct
gatttttagtgctattggggttgtaagttatatgggtttttggtttttgtgagggttatatatg
ggatgctgagttataggttgttcttatgggttcatttgatcattcatttgtctttgtatctatt
ggttccattccaatcatgtgcttttctgattttgaaggtggacttacttgacacagtttattca
gccttgacttctctcttttgctccttatttgatactttttttcctctttcatagaaagaatagtg
tttttaatgaatggttctgttctttgggttcttgacacttaacctcactgctgatttggtttct
atgttctgaccatgatatttgcttgttcccatgtcatctttattcttgcaatcatttttcttaac
tgggaattgctcacgtccctctgcatatttagttattggataggtgctgccttttgactactttg
ctcaactattttgttctcattcttttcaatcaagtgtgttcatgtacattaaaacgtgctgaat
tgatcgtgttcatatgaaagtgaaatttaggattggaggaaagtggaaatggaagtttgtgcta
tgatttggaactaccgccgtagcgctgacgtatctttgatatttgcttggttattggttcttat
tctcacttttgtttatgatttttgggtctgggctaccgaggcttagtggatagagatgataaac
tagggtcacctagtggtggaggtacaatctgcatctgaattctgtgtactaggacagatgtctg
tatgtatatatatatatatatatatatatatattcttttctcatatccaagacaactttttgttc
catacttttgctcaagtccattgaaggcctctctatggagactctttattcttttctatcccgg
agataggggggtggagtggcatttaaattggtttccttgctgcctacctttttgttagcttacct
ttttgtggattctgagagtcatgatactttttttagttacgattctttttttaaccaccggtgcat
taatttaacctacaaattgagatttttatcttaaaatgcattttcagtgctttggtaatataag
atagtatcattttcgttctgttgcttagaactgacgtattgtacattgtggcagTTTATGCTTT
GTTAGGGTTCTTGGTTGGCAATGAGTGCACAGCCTAAATATAAGgtcagattctcctgccttta
catatatgaagatctaggaagtttttcatcataatttctagatgctttgagtcgcagCTTAAAG
AGA**ATG**

>*PanNF-YA6*$_{pro}$ (PanWU01x14_192330)
ATATGTCACTAGTCGCGTGCATGAGACTTTTTACAAAGACATTTTAAGCATGTTGCAAGTTAAA
AAAAAAAGGAAGAGACAATAATAATTTTATTTCATTTAAAAAGATAAATAAAAACAAATTTTAT
TTTAGTACAAAAGTTACTAATCTTTTTTGTGGTCATGATTTATGTGAATTTCTATAATAAACTC
AACTATGATTAGGATATATTGTTGTCTTTAATTTTTAAATCAATTCTTAGGAGAATTTATACAA
AATAACTCAAAATTTTCGTCTTTCTTTTATTTATGTTTTATTTTTCCCAAAAATTTTACATTGC
ACAAGTTTTTGTTTTCTTTTCTCTTTTTTATAGTTTATTACGTTATTACTATATAGTACTCAAA
TATTCAAAAAGTATATATATATATGGGTACCAATCCAGTAAGTATTTATTTTTACTTATACACAAG
TATCATTTTGACCAATAAAATTTTTTTTTTATATTAAAAAGTAAAGGAGAGAAATAATATTAAT
GATGCATGATTTTTCATGCACTTCATTATATAGTAGATTTTTTTTTAACCTATGTGTAGGTATT
TTTTTTAGCCGTTGGATCATCATCTAATGGTGATCTAATAGTTATAATGAAATGCATAAAAAGA
CATGCATCATTAATATTATTTCTCTCTTTTATTTTTTAATATAAAAAATATTATATTAAAAAGT

AAGAGAAAGAAGTAAAAATGTTATATATAAAACTTATATATTTATATATTCGCTTATATAAGTT
ATCAATCCAGTAGATATATTTTTTCCCTATGTGTAAGTATTTTTTTAACCGTTGGATCATCATT
TAATGGTGATCTAATGGCTATAATGAAGTACATGAAAATACGTACATTATTAATGTTATTTCTC
TTTCTTACTTTTAATATAAAAAAATGTGCAAGAAAAATTTTTATTGGCCAAAATGATACCTATA
TATAGATAAAAAAAATACCTACTAGATTGGTGCCTATATATATATATATATAATACATATCCCT
TATTTCAAATAGAAAAATTGCCAATTTATTTGATAAAAGCAAATTGCCCAAAATTTTTTTTCAA
ATCTACTTCAGTGTTACATACTCATTTCTAACTATTCCTAACAATAAAGAAATGATAAGGTATT
CACTCAGAAAAAAAAGAAAAGAAATGATAAGGTATAAAATGAGATACTCTCATAAATCGAGTGC
CAAGACAGAAATATTATAAAATTTTAGTCTTATGTATAATTCAAGAGCTAAGATAAGGATGTTT
TTTTATTTGTTTGTAATTGAAGTGTCACACAAGGAGTTAAATAAGATGGAAGAAAAACATCTGC
GTGCCAAAATGAGGACATTTTTGAAGTTTAGTTGTCAAAATAGGACTTGAATAATAATCCGGGA
TCTAAATATGGGATTAACCCCATATTCAGCTATTTTATATCTTAATTTTTCATCCATTTATTTA
TTTATTATCTGTTCCCCTTTGCCAGCTGCTGAGAGGACTTTCCTCTGACTGGGTCTGGTTGGGA
AAGGAAGATAAGGAAATATGTGAAAAATCTTTGGAGTTGGATATGTTCTAAGCAACTGAGCAGA
TCATTTCTTGGCCATGCCACGTGTATGGCATAGTTGCCCTGCGCTAACCAAATATATTAAACGA
GTCTCTCAAGTCATCCTACTACCCGACAACACAGGACGATAACGACAAATACCTTTTCTTTTCT
TTTCTTGTCTTTTCTTTTCCCTTTTTTTCTTTTCGTTCTCTTATTATTACCTACATCCTATTCA
TACCAATTTGTCTATTTACTCAAGAGCCCCACATTGCATCATACATTTACAACCACTGTAATAC
GTCACCAAAAAAAAAGGAAAACAATTTGTCTATATTACTGTTGTGCGATCTTGATATTGAGTCT
ACTTTATTAGTTAACATCGCATATTAACATTTTAGCCATAGATGCTACCTGATCCGAATAAAAA
ATATATGAACGGGCGATAAAAGAAAATTAGATGAAAATTTTAAATGGATACTTGTGAAGAAAAA
GAAACAAATTTATTATAAAGATCAAATAATTTATTTATTAGTTGTTATTTAATTTTATATTGAT
ATTTAAAATTATATTTTCTGATTATGTAATTTTGTATTATTATTCAAAATTATATCTTCTAATT
ACTTTCTTGTTTTCGTAATAAAAAATATTGAAGTACCCTCGACTATATTCTTACATTTTTATTT
TGTTTTATTATTAAATTCATTTTCACACACAAATTTAATCTAATACATCATCACATTTTTCTCT
CCTCTCTTTTGATTTATTGAGAAAGAAAAAGAGCTAATTATAAATCTGATATACTTCGTGGGGA
AATATACTGCAAAATCAATTGAATCGAAACCTTCCAATGAAAAAGGTTAAAAATAAAGGTATGA
CTTCATAAGAAATTCTTTTTCTGTAGTAACCTTTCTTTAATTTCTTTTTTTTTTAGTTATGTCAT
GCAAATTACTCGTGGTCCAAATGAACTCCAAGATTATACGTCGTTTTTCTCTAATTATTGTCGG
TGAGTTAGTGCTGGGTATTTCCGAAAATAAACTAACAATTCCGGGGCCAATACTAGTATATACG
CGACGATAAGTTCAGGCAGCATTCTAAGTCACCAGAGAGGAGAGTGACGAACACCGCCAATCAG
AAAACACGAAGGCCTCGGAATTTGGATCGAAAGTGATTGGTCGGAGGGATCGGTTGGGAGTGAA
GCGTCAGGATCCGAGCGTGGGCCGAGAAGTGTTGACACAGTGAAGGGCGGCTGACAACAGAGA**G**
ACCGAATGAAGCCAAAAGGGTCTTAGCTCTTAAGGCCGTGTTTGGACGTGACTCGTGGGCCCCA
CCCAGAGAAAGACGTTAAATCATAAAATATAATTTGATTTTTTAAAAAACAAAAGCCAGTAGTG
AATCACGACAGACAGAGAGAGAGAGAGAGAGAGAGTCGGTAAAAGTGGTGGTGGGCAGTGGGTG
TTTTTATTGGGGAATTGAATTAAAATGGTTTTTTTTTTTTTGTATTATTATTTACAGCAAACCC
AAGAAGAAGATGATGAGAAGAGAAGGGAGAGAGAGAGAGTAAAATATAGATAGAGAAAGAGAAG
GAAGAGGAGACATGTCCAAGTTCATCCCAACAGCTCTGTAGCTTGCTCCTTCATAGCGTATCAG
ATTCTATTCCAAAGgtc**g**ctctttctcgcttctttttttctttttttcctttatttatttgttat
tattctttctaatcccgtccatctccactagtttcccacctttcccccttttttttcttttaatg
agtggatttctcgtggggttcatttattggtgtagaatataagtgggttttgttctatttgctg
tttagcgagtccatatatacagagtctgagatcattgtggtgtttgttgaggaagggaaagtgg
tcctggtaaagtctttttatggatgatgttggttttctcattctggccttatccctgttttgga
tcttctgctttttcagttcttggttttgggtttatcatatcttttgccatttcatcttttatct
ttttaggtttatgggtattttcattttttttttttactgcatttggttgaaaataaatggatgtg
tttatatttgtataaagtttggggcttaaggctttaattagtatgttaaattgttaatatgggt

ggtttgtgcttatttatttgtgggtttcatagttgatttcttaagcgatctgctagtttgagct
ttttctgttgatttggacatatactgcatgtaatgttatctttcttgcccactgaaccataggt
ctttttacttgtaatctgggttttgtctaggtacttttttgagaaaataatcaacaattttggaa
aaaattttgtaccctttaagttttacatcttgatgtgtgcctttaagtagtagctttgtgatg
tttgtatgttacatgtggttgtttggttatgtcactgaattttgtacgcagcaagttttggagt
caattattgtgcttgtgggatttcttgtttagtctgctttcttgctggactagtgcttatgaaa
caatggcacgttacctaatctgttattgaaagagctagaataggaaatgtttaaaggaaacaaa
aaggagaattatgaaaaggaatgcttgatatctattttagctgtttattaatacttattatgtg
taggactcacataatctctttgctaaatttgtaagtaaggacattaatgaataaaggaaggaaa
tactccctttcattctctttccatttattacacggaaacatgttatagatatgatagattttt
tttaaatcaattttaactctcaattgggacatgcctgtactctgtgcatggtagtacgcgtatg
cttacatacatacatacatgcatacatacatactatcttggttggcaactataaaaatacattc
tctcttaagatatggagacagaaatagctcattttatgttatcttccaattttggacttttatt
atattcctgttatttttatcttggggattgattgtttatattgactagattactctttgctatt
gaaacttgcccaacaaaagtgtcattccgagataattaagttagcaccttttttaccattaatt
tccctcaaatttcaGCAAATTCCAGACTAAATGTAAGCTTCATAGTGGAAGTTCAACTGGATCT
TCATCAACGCGTTACTAAGCATTTAGGGTTTCAAATTTGGAGTACTTTAGAAGGAAACATCTAC
AACACGAGCAATTGGTCAATCTCAAGAAAATTTGTGGTTTGGGAGGGACCAATTAAAAAGATCC
ATG

Table S4: Gene identifiers for NF-YA proteins used to build the phylogenetic tree depicted in Figure 4 and Figure S7.

Name	Gene Identifier
AtNF-YA1	AT5G12840
AtNF-YA2	AT3G05690
AtNF-YA3	AT1G72830
AtNF-YA4	AT2G34720
AtNF-YA5	AT1G54160
AtNF-YA6	AT3G14020
AtNF-YA7	AT1G30500
AtNF-YA8	AT1G17590
AtNF-YA9	AT3G20910
AtNF-YA10	AT5G06510
Fve_mrna07522	mrna07522.1-v1.0-hybrid
Fve_mrna12709	mrna12709.1-v1.0-hybrid
Fve_mrna14681	mrna14681.1-v1.0-hybrid
Fve_mrna18295	mrna18295.1-v1.0-hybrid
Fve_mrna26819	mrna26819.1-v1.0-hybrid
Fve_mrna28941	mrna28941.1-v1.0-hybrid
Gma_02G195000	Glyma.02G195000
Gma_02G303800	Glyma.02G303800
Gma_03G203000	Glyma.03G203000
Gma_05G166100	Glyma.05G166100
Gma_07G036200	Glyma.07G036200
Gma_08G124200	Glyma.08G124200
Gma_08G335900	Glyma.08G335900
Gma_09G023800	Glyma.09G023800
Gma_09G068400	Glyma.09G068400
Gma_10G082800	Glyma.10G082800
Gma_12G236800	Glyma.12G236800
Gma_13G107900	Glyma.13G107900
Gma_13G202300	Glyma.13G202300
Gma_14G010000	Glyma.14G010000
Gma_15G027400	Glyma.15G027400
Gma_15G129900	Glyma.15G129900
Gma_15G173300	Glyma.15G173300
Gma_16G005500	Glyma.16G005500
Gma_17G051400	Glyma.17G051400
Gma_18G071000	Glyma.18G071000
Gma_19G200800	Glyma.19G200800
Lj_FS318732	FS318732.1
LjNF-YA1	Lj5g3v0841080
LjNF-YA2	Lj6g3v0647470

LjNF-YA3	Lj4g3v2179250
LjNF-YA4	Lj1g3v4752710
LjNF-YA5	Lj3g3v2657800
LjNF-YA6	Lj3g3v0338970
LjNF-YA7	Lj2g3v3336090
LjNF-YA8	Lj0g3v0252369
MnoNF-YA1	XP_010102352.1
MnoNF-YA3	XP_010087689.1
MnoNF-YA7	XP_010098569.1
MnoNF-YA8	XP_010090113.1
MnoNF-YA9a	XP_010105454.1
MnoNF-YA10	XP_010106984.1
MnoNF-YA9b	XP_010088228.1
MtNF-YA1	Medtr1g056530
MtNF-YA2	Medtr7g106450
MtNF-YA3	Medtr2g041090
MtNF-YA4	Medtr2g099490
MtNF-YA5	Medtr3g061510
MtNF-YA6	Medtr2g030170
MtNF-YA7	Medtr8g037270
MtNF-YA8	Medtr8g019540
PanNF-YA1	PanWU01x14_284830
PanNF-YA2	PanWU01x14_161830
PanNF-YA3	PanWU01x14_246880
PanNF-YA4	PanWU01x14_050420
PanNF-YA5	PanWU01x14_192760
PanNF-YA6	PanWU01x14_192330
PanNF-YA7	PanWU01x14_231390
Ppe_2G014500	Prupe.2G014500
Ppe_3G043900	Prupe.3G043900
Ppe_4G090200	Prupe.4G090200
Ppe_4G249900	Prupe.4G249900
Ppe_7G093100	Prupe.7G093100
Ppe_7G203500	Prupe.7G203500
PvNF-YA1	Phvul.001G196800
PvNF-YA2	Phvul.002G246600
PvNF-YA3	Phvul.005G156100
PvNF-YA4	Phvul.003G133100
PvNF-YA5	Phvul.008G283100
PvNF-YA6	Phvul.011G211300
PvNF-YA7	Phvul.006G062200
PvNF-YA8	Phvul.010G133300
PvNF-YA9	Phvul.007G267100
TorNF-YA1	TorRG33x02_341480
TorNF-YA2	TorRG33x02_150260

TorNF-YA3	TorRG33x02_081410
TorNF-YA4	TorRG33x02_339730
TorNF-YA5	TorRG33x02_031550
TorNF-YA6	TorRG33x02_321500
TorNF-YA7	TorRG33x02_125390
AduNF-YA1	XP_015951283
AduNF-YA2	XP_015946278
AduNF-YA3	XP_015954718.1
AduNF-YA4	Aradu.67X2R
AduNF-YA5	XP_015972671.1
AduNF-YA6	XP_015956129.1
AduNF-YA7	XP_015937254.1
AduNF-YA8	XP_015935517.1
AduNF-YA9	XP_015959290.1
Dgl3616S29258	Dgl3616S29258
Dgl216S24617	Dgl216S24617
Dgl81S35216	Dgl81S35216
Dgl959S36562	Dgl959S36562
Dgl402S29919	Dgl402S29919
Dgl544S31934	Dgl544S31934
Dgl749S12860	Dgl749S12860
Cgl33S15932	Cgl33S15932
Cgl26S24714	Cgl26S24714
Cgl31S04874	Cgl31S04874
Cgl428S01957	Cgl428S01957
Cgl231S11946	Cgl231S11946
Cgl8S22241	Cgl8S22241
Cgl24S00922	Cgl24S00922

REFERENCES

van Velzen R, Holmer R, Bu F, Rutten L, van Zeijl A, Liu W, Santuari L, Cao Q, Sharma T, Shen D, *et al.* 2018. Comparative genomics of the nonlegume *Parasponia* reveals insights into evolution of nitrogen-fixing rhizobium symbioses. *Proceedings of the National Academy of Sciences of the United States of America* **115**: E4700–E4709.

Soyano T, Kouchi H, Hirota A, Hayashi M. **2013**. Nodule inception directly targets NF-Y subunit genes to regulate essential processes of root nodule development in *Lotus japonicus*. *PLoS genetics* **9**: e1003352.

Soyano T, Shimoda Y, Hayashi M. **2015**. NODULE INCEPTION antagonistically regulates gene expression with nitrate in *Lotus japonicus*. *Plant & cell physiology* **56**: 368–376.
